# Genomes from Uncultivated Pelagiphages Reveal Multiple Phylogenetic Clades Exhibiting Extensive Auxiliary Metabolic Genes and Cross-Family Multigene Transfers

**DOI:** 10.1128/msystems.01522-21

**Published:** 2022-08-16

**Authors:** Fabian Wittmers, David M. Needham, Elisabeth Hehenberger, Stephen J. Giovannoni, Alexandra Z. Worden

**Affiliations:** a Ocean EcoSystems Biology Unit, RD3, GEOMAR Helmholtz Centre for Ocean Research Kiel, Kiel, Germany; b Kiel University, Kiel, Germany; c Department of Microbiology, Oregon State University, Corvallis, Oregon, USA; d Marine Biological Laboratory, Woods Hole, Massachusetts, USA; e Max Planck Institute for Evolutionary Biology, Plön, Germany; University of Hawaii at Manoa

**Keywords:** DNA Hypermodification, phage aggregation, horizontal multigene transfer, SAR11 life cycles, single cell metagenomics, T8SS, Curli fibers, thymidylate synthase, auxiliary metabolic genes, Pelagiphage

## Abstract

For the abundant marine Alphaproteobacterium *Pelagibacter* (SAR11), and other bacteria, phages are powerful forces of mortality. However, little is known about the most abundant Pelagiphages in nature, such as the widespread HTVC023P-type, which is currently represented by two cultured phages. Using viral metagenomic data sets and fluorescence-activated cell sorting, we recovered 80 complete, undescribed Podoviridae genomes that form 10 phylogenomically distinct clades (herein, named Clades I to X) related to the HTVC023P-type. These expanded the HTVC023P-type pan-genome by 15-fold and revealed 41 previously unknown auxiliary metabolic genes (AMGs) in this viral lineage. Numerous instances of partner-AMGs (colocated and involved in related functions) were observed, including partners in nucleotide metabolism, DNA hypermodification, and Curli biogenesis. The Type VIII secretion system (T8SS) responsible for Curli biogenesis was identified in nine genomes and expanded the repertoire of T8SS proteins reported thus far in viruses. Additionally, the identified T8SS gene cluster contained an iron-dependent regulator (FecR), as well as a histidine kinase and adenylate cyclase that can be implicated in T8SS function but are not within T8SS operons in bacteria. While T8SS are lacking in known *Pelagibacter*, they contribute to aggregation and biofilm formation in other bacteria. Phylogenetic reconstructions of partner-AMGs indicate derivation from cellular lineages with a more recent transfer between viral families. For example, homologs of all T8SS genes are present in syntenic regions of distant Myoviridae Pelagiphages, and they appear to have alphaproteobacterial origins with a later transfer between viral families. The results point to an unprecedented multipartner-AMG transfer between marine Myoviridae and Podoviridae. Together with the expansion of known metabolic functions, our studies provide new prospects for understanding the ecology and evolution of marine phages and their hosts.

**IMPORTANCE** One of the most abundant and diverse marine bacterial groups is *Pelagibacter*. Phages have roles in shaping *Pelagibacter* ecology; however, several Pelagiphage lineages are represented by only a few genomes. This paucity of data from even the most widespread lineages has imposed limits on the understanding of the diversity of Pelagiphages and their impacts on hosts. Here, we report 80 complete genomes, assembled directly from environmental data, which are from undescribed Pelagiphages and render new insights into the manipulation of host metabolism during infection. Notably, the viruses have functionally related partner genes that appear to be transferred between distant viruses, including a suite that encode a secretion system which both brings a new functional capability to the host and is abundant in phages across the ocean. Together, these functions have important implications for phage evolution and for how Pelagiphage infection influences host biology in manners extending beyond canonical viral lysis and mortality.

## INTRODUCTION

The most abundant bacteria in the surface ocean are minimalistic, free-living heterotrophs belonging to the SAR11 alphaproteobacterial lineage, *Pelagibacter* (1, 2). Although death by viral lysis is an important force of mortality for marine bacteria, knowledge of the diversity and biology of phages infecting SAR11 lineages is nascent. Cultured representatives are still sparse (3, 4), in part due to the challenges behind culturing both SAR11 hosts and their phages, although progress is being made in this regard (4, 5). To date, cultured Pelagiphages come from three families: Myoviridae, Podoviridae, and Autographiviridae (6). Cultivation-independent metagenomic studies have been used to more fully characterize the genomic content of Pelagiphages and the potential molecular mechanisms by which they manipulate hosts and their ecology (7). The latter have contributed to an expanded view of core genomic attributes, particularly for the Myoviridae and the Autographiviridae HTVC010P-type Pelagiphages. Additionally, the identification of auxiliary metabolic genes (AMGs) has expanded phage-host ecology studies from focusing purely on the viral life cycle and host mortality to also considering how specific phage genes repurpose, manipulate, and/or augment host metabolism during infection (8).

AMGs often encode critical proteins of host metabolic pathways that act as bottlenecks for viral propagation during infection (8–14). Perhaps the best studied group of marine phages with respect to AMGs are those that infect the Cyanobacteria *Prochlorococcus* and *Synechococcus*. For example, Cyanophage photosystem genes appear to have been acquired independently from contemporary hosts multiple times (15, 16), with some Cyanophages having just psbA, some just psbD, and others having both genes (17). The availability of cultures of these phages has facilitated the demonstration of the fact that host synthesis of virally encoded psbA and/or psbD results in an augmentation of photosynthesis (18). In some cases, viral AMGs cannot be traced to extant hosts at all (17), whereas in other cases, for example, cyanophage transaldolase C protein sequences in cultured Myoviridae and Podoviridae (9, 19), greater homology exists between those of the two viral families than to host versions, suggesting between-virus transfers (9, 19).

With respect to Pelagiphages, metagenome-based studies have revealed not only the diversity of uncultivated Pelagiphages but also AMGs and variable regions that they carry (7, 20). This is particularly true for the Myoviridae, for which a survey of 26 viral metagenome-assembled genomes from phages that putatively infect *Pelagibacter* (7) identified two main groups of functionally related AMGs. One group contained genes associated with nucleotide metabolism, such as cobalamin synthases (cobS, cobT) and peptide deformylase (PDF), and the other group contained genes associated with lipopolysaccharide modification (glycosyltransferases). The same study (7) also reported a partial Type VIII Secretion System (T8SS, also known as Curli) which, in bacteria, is implicated in adhesion, aggregation, and biofilm formation (21). Specifically, they found a clustered region that includes the known T8SS genes CsgF and CsgG, as well as two hypothetical proteins and another gene encoding an unspecified “Curli-associated protein”. Although two other known essential T8SS proteins were not explicitly identified, these types of searches provide valuable starting points for considering the repertoire of genes encoding metabolic functions that might shape *Pelagibacter* physiology, once infected. Moreover, they raise questions regarding the taxonomic origins, acquisition, and retention of AMGs that are both colocated in the phage genome and functionally related, herein termed partner-AMGs.

To date, knowledge levels are uneven between the different families of Pelagiphages, with those considered the most abundant in nature being less well-characterized than others. Members of the Podoviridae and Autographiviridae that infect SAR11 are considered highly abundant (22), especially the Podoviridae HTVC023P-type Pelagiphages (6). However, thus far, just two HTVC023P-type Pelagiphages have been cultured and genome sequenced (6), and both were isolated against the same SAR11 isolate (HTCC1062). Thus, there is still much to be learned about the potential gene content of the HTVC023P-type Pelagiphages, including uncultured relatives, such as those represented by partial environmental genome assemblies that were noted (6) in the GOV2.0 database (23). Here, we address three topics regarding the HTVC023P-type lineage of Podoviridae. First, we examine levels of phylogenetic and genomic diversity that can be recovered from ocean data and assembled into complete genome sequences of environmental HTVC023P-type relatives. Second, we characterize the core and AMG repertoires of these viruses. Third, we perform phylogenetic studies to test for the taxonomic origins of retained AMGs that are functional partners. For these purposes we used fluorescence-activated cell sorting (FACS) at sea, systematic queries of global reference environmental viromes (23), tailored assembly methods, and robust phylogenetic methods to identify a suite of uncultured Pelagiphages belonging to the Podoviridae. Collectively, we phylogenomically relate multiple circular, complete “wild” HTVC023P-type genomes and analyses of these provide new insights into the HTVC023-type lineage. Additionally we identify an unprecedented gene cluster transfer of partner-AMGs between the Myoviridae and Podoviridae families, suggesting a strong selective pressure for the retention of an entire “viral” T8SS and the function it encodes.

## RESULTS

### Recovery of genomes from uncultivated phage of interest.

We used a multifaceted approach to recover the complete genomes of uncultivated Pelagiphages of the HTVC023P-type Podoviridae. This started with BLASTP searches using the DNA polymerase A (PolA) gene from Pelagiphages HTVC023P and HTVC027P. Searches were performed against genome assemblies from bacteria FACS sorted from the Pacific Ocean that then underwent whole-genome amplification using multiple displacement amplification (MDA), sequencing, and assembly. Searches were also performed against published metagenomic contigs from a FACS-based bacterial study (24) and metagenomic virome contigs from the GOV2.0 database (23). The recovered viral contigs encoded PolA genes that overlapped with some of those identified in (6) as putatively being from the HTVC023P-type, and were then all evaluated for completeness. We were able to circularize a subset of these using terminal overlaps. These efforts rendered 81 environmental circularized viral genomes (envCVGs) of putative Pelagiphage for subsequent analyses (Data Set S1A-I).

10.1128/msystems.01522-21.1 DATA SET S1 General features, Curli sequences, ortholog analysis, GOV2.0 Curli frequencies, and metatranscriptome read recruitment of complete HTVC023P-type genomes. Download Data Set S1, XLSX file, 0.6 MB. Copyright © 2022 Wittmers et al. 2022 Wittmers et al. https://creativecommons.org/licenses/by/4.0/ This content is distributed under the terms of the Creative Commons Attribution 4.0 International license.

### Phylogenetic identification and core characteristics of uncultivated HTVC023-type genomes.

**FIG 1:**
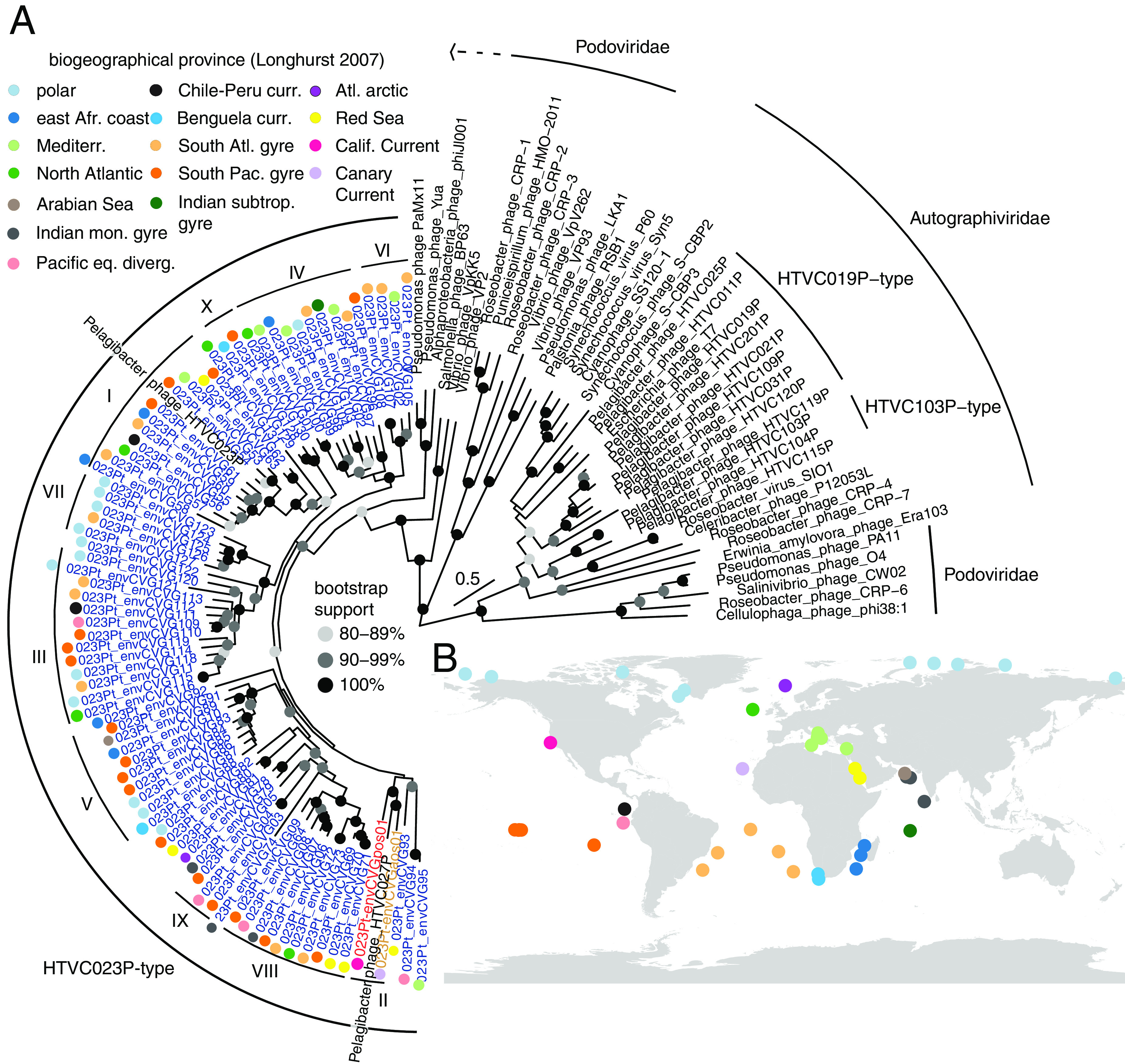
Evolutionary relationships between Podoviridae and the recently partitioned Autographiviridae (e.g., Pelagiphage HTVC019P-type) and their distribution across biogeographical provinces. (A) Maximum likelihood (ML) reconstruction of PolA under the LG+I+G4 model. Sequences named “*Pelagibacter* phage” come from cultured phages shown to infect one or more *Pelagibacter* isolates, whereas sequences labeled “env” represent PolA from envCVGs (Data Set S1D). POS and AOS indicate PolA from circularized phage genomes derived from at-sea FACS and subsequent sequencing. Node support reflects the percentage of 1,000 ultrafast bootstrap replicates. (B) Map with all stations queried. Colors indicate the biogeographic province of recovery, as included in (A), based on Longhurst et al. (25). HTVC023P-clades were assigned based on supported phylogenetic clades.

To investigate the true relatedness of the 81 envCVGs to one another and to other viral lineages, we performed a maximum likelihood reconstruction that incorporated PolA from the envCVGs, Podoviridae, and Autographiviridae lineages, including cultured isolates of the HTVC023P-type (Podoviridae) and the Autographiviridae HTVC019P-type and HTVC103-type Pelagiphage lineages. Viral lineages that lack PolA (and instead have PolB), such as Myoviridae (6, 7), were excluded. The analysis indicated that 68 of the newly identified envCVGs belonged to two statistically supported groups, with two Podoviridae of Pseudomonas hosts and an alphaproteobacterial phage placed in an outgroup position (6). One of the supported groups contained the two cultured HTVC023P-type phages, HTVC023P and HTVC027P, as well as FACS-derived 023Pt_envCVGaos01 and 023Pt_envCVGpos29 (for “Atlantic Ocean Sort” and “Pacific Ocean Sort”, respectively) and many other envCVGs, while the other group contained 13 envCVGs (Fig. 1A). A multigene analysis was also performed, in this case using a concatenated alignment of five conserved proteins (PolA, DEAD/DEAH box helicase, primase, capsid, and one hypothetical protein [Fig. S1]). Although the backbone of the resulting reconstruction differed from that of the PolA reconstruction, the general topology, in terms of clade structuring, was similar. Hence, we delineated and named 10 clades (I to X) that were coherent between the two trees (same members) and had three or more members, with the requirement that the clade retain ≥90% bootstrap support in both reconstructions (Fig. 1; Fig. S1). In both trees, 023Pt_envCVG74 exhibited a long branch, and in the multigene tree, it was placed outside the overall Pelagiphage group. Therefore, we do not term it as a putative 023Pt Pelagiphage, reducing the final set to 80. Clear correspondence was not observed between either the biogeochemical provinces (25), or geographic locations, where the phage genomes were recovered and the phylogenetic clade structure.

10.1128/msystems.01522-21.1 FIG S1 Maximum Likelihood multigene phylogeny of HTVC023P-type Pelagiphages and related environmental phage genomes. The alignments are based on PolA, DEAD/DEAH box helicase, primase, capsid, and one conserved hypothetical with phylogeny computed using the LG+C20+G+F model. The tree was rooted for visualization purposes based on Pseudomonas phage PaMx11 and Alphaproteobacteria phage phiJL001, the closest known relatives of the HTVC023P-type (6). Phage 023Pt_envCVGaos01 came from an Atlantic Ocean Sort (24), in which we identified the sorted bacterium as being *Pelagibacter*. Node support reflects the percentage of 1,000 ultrafast bootstrap replicates under the LG+C20+I + G+F mixed model. Written bootstrap support on selected nodes reflects node support under for a second phylogeny run under the LG+F+I+G4 model. Download FIG S1, EPS file, 2.2 MB. Copyright © 2022 Wittmers et al. 2022 Wittmers et al. https://creativecommons.org/licenses/by/4.0/ This content is distributed under the terms of the Creative Commons Attribution 4.0 International license.

The 80 putative HTVC023P-type envCVGs came from 39 stations, spanning five major seas and oceans (Fig. 1B), highlighting the broad geographic distribution of this Pelagiphage lineage that has been observed in previously single-gene environmental analyses (6, 26). On average, all HTVC023P-type envCVGs have similar sizes; with a mean genome size of 58,776 ± 2,523 kb and 42.1 ± 6.4% G+C content (*n* = 82, which includes the cultivated HTVC023P and HTVC027P). These phages encoded, on average, 82 ± 9 open reading frames (ORFs) per genome.

**FIG 2:**
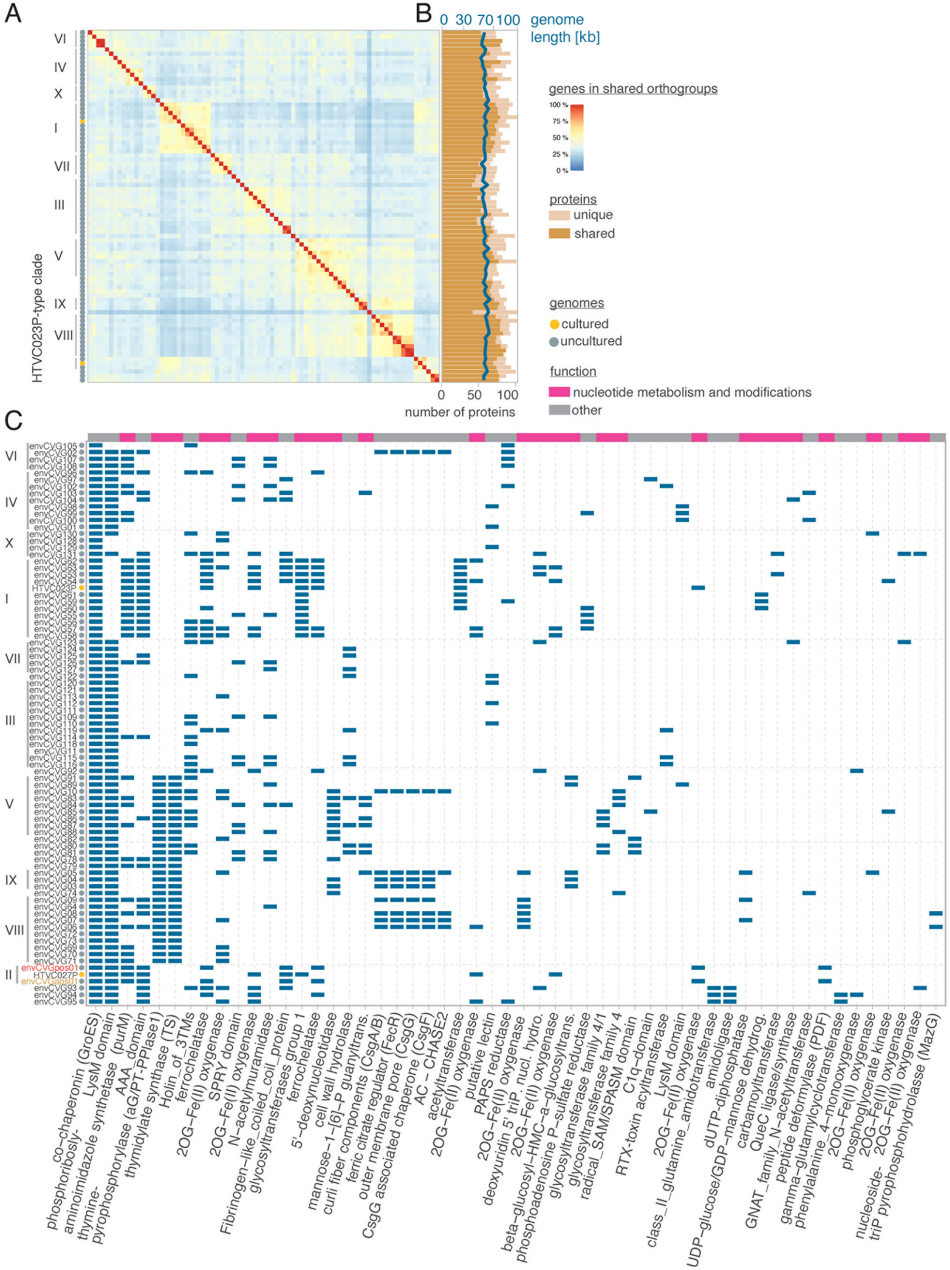
Orthogroup content patterns connect to phylogenetic relationships with patchier distribution of AMGs across HTVC019P-type and env102 clades. (A) All-vs-all pairwise comparison of Pelagiphages showing the percent of orthogroups that are shared between given Pelagiphages. The heatmap is sorted by the phylogenomic results as in Fig. 1A, thus demonstrating the degree to which phylogenetic clades have shared proteins. (B) Genome size and number of shared and unique proteins for each HTVC023P-type Pelagiphage. Proteins were classified as shared if they had an ortholog in at least 1 other HTVC023P-type genome. Unique proteins refer to proteins with one or more copies within a single one of these genomes but no detection in the others. (C) Presence or absence of all AMGs found in at least two HTVC023P-type phage genomes. Annotations are based on a BLASTP analysis against NCBI nr (*e*-value < 1 × 10^−10^) and hmmsearch against Pfam (*e*-value < 1 × 10^−10^) and were manually curated. For all panels, HTVC023P clades are assigned according to the results of Fig. 1A.

Protein clustering revealed that the HTVC023P-type pan-genome contains 2,007 genes, 79.8% of which belonged to orthogroups (Data Set S1A). The core genome had 13 genes and consisted of viral replication and DNA packaging genes (helicases, terminase, and DNA polymerase), a co-chaperonin GroES, and eight other genes with no annotation (Data Set S1I). Across all HTVC023P-type genomes 623 orthogroups were identified, that is, groups that contained orthologs present in two or more genomes. On average, 17 ± 9 unique (i.e., not found in another HTVC023P-type phage) genes were observed per genome (Fig. 2A and B; Data Set S1A). The distribution of orthogroups within the phage genomes corresponded well with the clade structure of the HTVC023P-type lineages (Fig. 1A and 2A). These results suggest that evolutionary relatedness, as resolved in clade structure, connected with ortholog content and that the acquisition and retention of these orthologs/orthogroups was relatively independent of the geographic location where they were observed in today’s oceans.

### Auxiliary metabolic gene distributions.

Functional annotation of the 623 orthogroups identified in the uncultured HTVC023P-type phages, as well as the two cultured representatives, were examined using identified Pfam domains and similarities to potential homologs in NCBI nr. 569 of the orthogroups could not be further classified because they lacked identifiable Pfam domains or were too distant from known sequences to allow for inferences regarding possible functions. However, 54 were assigned putative cellular functions and showed homology to sequences from Bacteria, Archaea, or eukaryotes, and were termed AMGs herein (Fig. 2C). This AMG number is four-fold higher than that previously reported (13) from the genomes of isolates HTVC023P and HTVC027P (6).

Importantly, the new AMGs (for this lineage) highlighted a breadth of putative functions, with genes encoding proteins for DNA or protein modifications being most prevalent (Fig. 2C). 45 percent of the 82 genomes from the Podoviridae Pelagiphage lineage carried a phosphoriboslyaminoimidazole synthetase (purM), involved in purine biosynthesis (27), which was often associated with an AAA-domain. Clade II 023Pt_aos01 and 023Pt_pos29 encoded peptide deformylase (PDF), which catalyzes an early reaction of purine biosynthesis (Fig. S2) (22). We also identified three glycosyltransferases, proteins which can be involved in the modification of DNA or proteins (7). Additionally, members of the diverse 2-oxoglutarate (2OG) and iron(Fe[II])-dependent oxygenase (2OG-Fe[II]) superfamily that were detected catalyze the incorporation of O_2_ into a range of molecules (28).

10.1128/msystems.01522-21.2 FIG S2 Biochemical pathway for purine biosynthesis in bacteria. Enzymes colored in green represent AMGs identified in at least one HTVC023P-type genome, highlighting the role of peptide deformylase (PDF) and phosphoribosylaminoimidazole-synthetase (purM). Overall pathway based on (73). Download FIG S2, PDF file, 0.1 MB. Copyright © 2022 Wittmers et al. 2022 Wittmers et al. https://creativecommons.org/licenses/by/4.0/ This content is distributed under the terms of the Creative Commons Attribution 4.0 International license.

**FIG 3:**
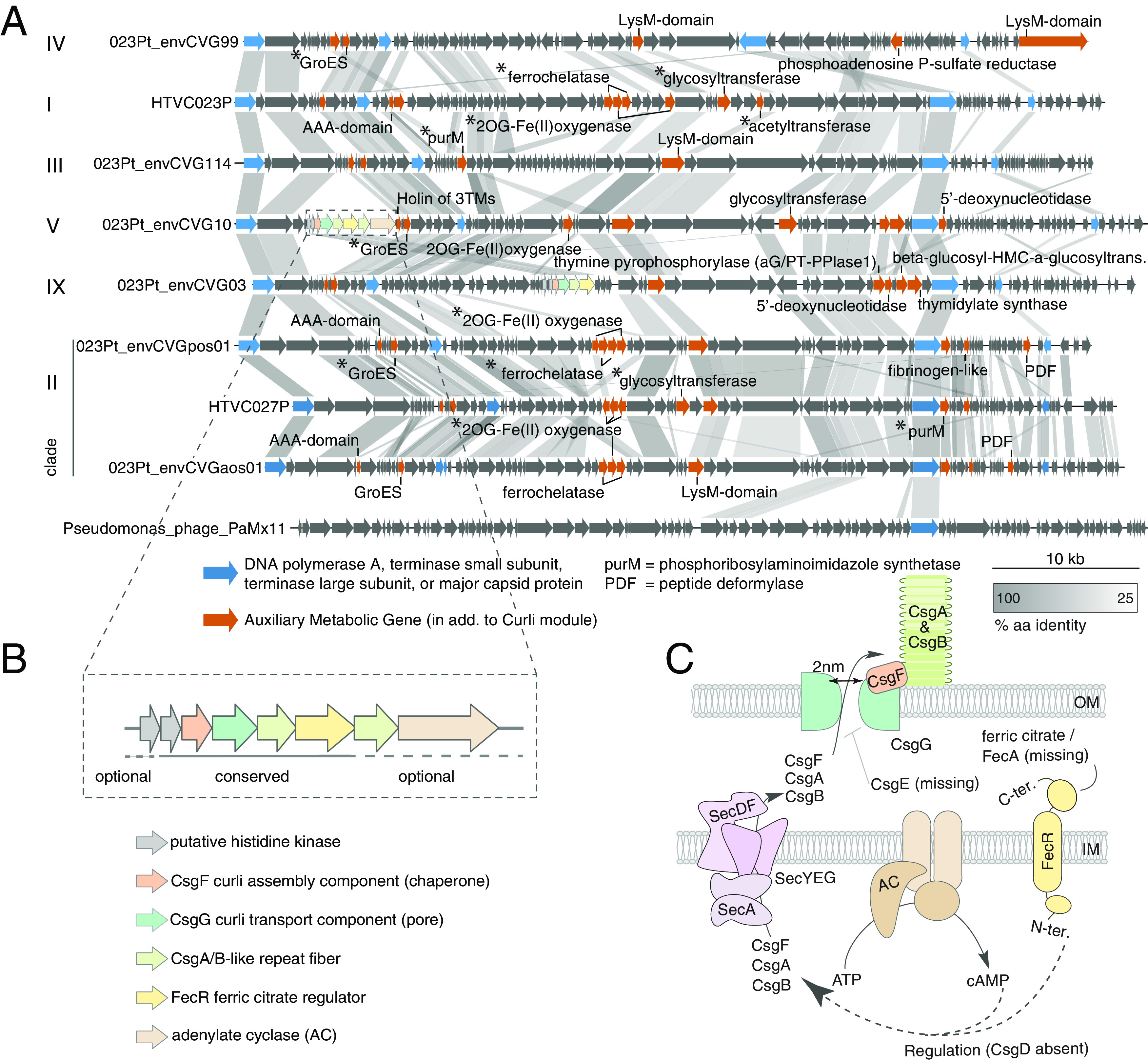
Genome synteny across Pelagiphage HTVC023P-type and the conservation of T8SS gene structure. (A) Linearized alignment of representative (circular) genomes from Podoviridae HTVC023P-type Pelagiphages along with related environmental sequences. ORF orientation (arrowheads) and homologous ORFs (shaded by amino acid similarity levels) are indicated. Curli genes are colored as in the enlarged module shown in (B). AMGs (identified in Fig. 2C) are indicated (orange), as are conserved core phage genes (blue). AMGs previously annotated (6) in the HTVC023P-type lineage are marked (*). (B) General organization of the viral Curli genes in 9 representative Pelagiphage Podoviridae circularized genomes. (C) Proposed cellular localization of the viral genes when expressed and translated in *Pelagibacter*, based on concepts from E. coli (30). Note that SecYEG, necessary for translocation proteins, including Curli components, into the periplasm, is encoded outside Curli operons in model bacteria (73) and is encoded in the *Pelagibacter* genome (Acc.: GCA_012276695.1).

Three AMG-types were notable for being colocated and for appearing to involve one or more functionally related AMG, in addition to potential host components that might be needed for their function. These three were the aforementioned 2OG-Fe(II) superfamily proteins, as well as DNA hypermodification enzymes, and secretion system related genes. The first of these involved eight distinct (nonorthologous) AMGs annotated as belonging to the 2OG-Fe(II) oxygenase superfamily. These were frequently nearby with in a genome (although not adjacent) and were also often colocated with a partner in the form of one or two ferrochelatases (Fig. 3A), enzymes responsible for the insertion of divalent iron cations into tetrapyrrole structures, such as Heme. *Pelagibacter* isolates are known to have an iron response regulator (29) acting on the expression of iron-dependent genes, with the regulator directly interacting with ferrochetalase. While gene ordering varied, whenever a phage encoded ferrochelatase, a 2OG-Fe(II) oxygenase was found adjacent to it (Fig. 3A). Note that the converse is not necessarily true as the oxygenases themselves could also occur as single entities.

The second AMG-type that involved partners had functions in DNA hypermodification (30) and was present in all Clade V, VIII, and IX HTCV023P-type Pelagiphages (Fig. 2C). Specifically, proteins encoding thymidylate synthase (TS) and alpha-glutamyl/putrecinyl thymine pyrophosphorylase clade 1 (aG/PT-PPlase1) were located nearby to each other (Fig. 2C and 3A). Additionally, a 5′-deoxynucleotidase and a putative beta-glucosyl-HMC-alpha-glucosyl-transferase were present in the same genomic region as TS and aG/PT-PPlase1 in multiple Clade II Pelagiphages, close to the DNA polymerase (Fig. 3A).

Finally, we noted multiple proteins involved in a T8SS in the envCVGs. The T8SS were in the genomes of four Clade VIII HTVC023P-type Pelagiphages and in three of which were in three members of the its sister clade, Clade IX. Single representatives of Clades V and VI also had the T8SS. These genes appeared not only to be colocated but also to have conserved order, unlike the above partner-AMGs (Fig. 3A and B). The Podoviridae T8SS region appeared to be functional (Fig. 3C) and encoded up to seven proteins, comprising on average 7.8 ± 1.6% of the entire phage genome. Three known T8SS genes were universally present: Curli assembly chaperone (CsgF), Curli transport pore (CsgG), and Curli repeat fiber (CsgA/B). These were always preceded by a histidine kinase (HK), sometimes two, and were followed by a ferric citrate regulator (FecR). The latter was frequently followed by a second Curli repeat fiber gene and an adenylate cyclase (AC), catalyzing cyclic AMP production.

Next, we utilized HMM-models built from the viral sequences identified herein to reexamine the Myoviridae MAGs, in which CsgG and CsgF had been explicitly identified, and for which other related proteins had been inferred to be present (7). Our searches exposed all of the T8SS genes essential for Curli fiber secretion, alongside the newly identified, putatively regulatory FecR and AC, including proteins that encode the critical Curli fiber repeats (CsgA/B) in the Myoviridae Pelagiphages (Fig. 3 and Fig. S3).

10.1128/msystems.01522-21.3 FIG S3 Conserved Curli operons in Myoviridae Pelagiphages. Shown are alignments of the complete Myoviridae HTVC008M-type Pelagiphage genomes recovered in (7). The gene predictions and subsequent annotations performed herein captured the complete Curli-encoding regions. Only phage genomes that could be circularized and that contain terminal overlaps are shown. ORF orientation (arrowheads) and homologous ORFs (shaded by amino acid similarity levels) are indicated, as are Curli genes (colored as in Fig. 3A) and highly conserved HTVC023P-type core genes (blue), as indicated on the figure. Download FIG S3, JPG file, 1.1 MB. Copyright © 2022 Wittmers et al. 2022 Wittmers et al. https://creativecommons.org/licenses/by/4.0/ This content is distributed under the terms of the Creative Commons Attribution 4.0 International license.

### Evolutionary origins of partner-AMGs.

We next pursued the potential origins of the partner AMGs, that is, those that were colocated and had related functions. In the case of the 2OG-Fe(II) superfamily members, the extent of the gene family expansion and short protein sequence length rendered the viral proteins unsuitable for phylogenetic analysis, precluding the rigorous pursuit of their potential origins in connection to those of their partner-AMGs. However, we were able to analyze both of the other types of partner-AMGs.

Of the two proteins involved in DNA hypermodification (Fig. 2C), we found that the HTVC023P-type aG/PT-PPlase1 sequences were placed in a broad clade of viral sequences (103 in total) seperate from orthologs from multiple bacterial lineages (Fig. S4). The Pelagiphage sequences formed a supported paraphyletic group that included sequences from marine environmental phages with unidentified hosts, and an interior clade which contained four sequences from PaMx11-like Siphoviridae. Adjacent to this broad group was a supported clade of sequences from M6-like Siphoviridae that infect Pseudomonas and *Bordetella*. Notably, all Clade V sequences grouped together in a supported clade, while the Clade VIII and IX sequences were distributed across the Pelagiphage group (apart from the PaMx11-like clade) and incorporated multiple other environmental sequences. Moreover, the topology of this reconstruction was akin to that of the phylogenomic reconstruction (Fig. S1) in that all three Clade IX sequences (envCVG_03-05), which came from two biogeochemical provinces in the South Pacific, were placed in a supported basal position to the broader group in which aG/PT-PPlase1 sequences from Clade V, VIII and IX members were placed (Fig. S4; Data Set S1H). In contrast, TS identified in 154 phages formed affiliations with several different bacterial lineages, while the Pelagiphage sequences belonged to a supported group containing 85 viral sequences and were in a region of the tree that also included sequences from unidentified environmental bacterial genomes, Gammaproteobacteria, and Cyanobacteria (Fig. S5). Apart from the most basal sequences, the backbone of this paraphyletic group of viral sequences did not retain support. However, again, Clade V sequences formed a supported clade that also contained other environmental phage sequences (unidentified hosts). As seen for the aG/PT-PPlase1, sequences from M6-like and PaMx11-like Siphoviridae were more closely related to the Pelagiphage versions than to other TS, based on currently available data.

10.1128/msystems.01522-21.4 FIG S4 Phylogenetic reconstruction of alpha-glutamyl/putrecinyl thymine pyrophosphorylase clade 1 (aG/PT-PPlase1). (A) Maximum likelihood reconstruction of viral aG/PT-PPlase1 and related sequences from the global earth microbiome (72) and NCBI nr. Branches are colored based on taxonomy, with metagenomic sequences that do not follow the dominant taxonomy of a branch being manually recolored. (B) Topology of the viral sequence branch in (A) at a higher resolution. Sequences originating from genomes described here are highlighted in red and orange. The phylogeny was reconstructed under the LG+R10 model with 1,000 ultrafast (UF) bootstrap iterations, using a total of 1,398 sequences and 383 amino acid positions. Branch support was omitted in (A) due to the high number of overlaying nodes, but it is available in the raw supplementary data. The extracted viral clade (B) had 85% UF bootstrap support. Note that Myoviridae, Siphoviridae, and Podoviridae are viral families of the Caudovirales order. Download FIG S4, EPS file, 2.7 MB. Copyright © 2022 Wittmers et al. 2022 Wittmers et al. https://creativecommons.org/licenses/by/4.0/ This content is distributed under the terms of the Creative Commons Attribution 4.0 International license.

10.1128/msystems.01522-21.5 FIG S5 Phylogenetic reconstruction of thymidylate synthase (TS) proteins. (A) Maximum likelihood reconstruction of viral thymidylate synthase and related sequences from the global earth microbiome (72) and NCBI nr. Branches were colored based on taxonomy, with metagenomic sequences that do not follow the dominant taxonomy of a branch being manually recolored. (B) Topology of the 100% bootstrap supported viral sequence branch in (A) at a higher resolution. Sequences originating from genomes described here are highlighted in red and orange. The phylogeny was reconstructed under the LG+R10 model with 1,000 ultrafast bootstrap iterations, using a total of 3,248 sequences and 211 amino acid positions. Branch support was omitted in (A) due to the high number of overlaying nodes, but it is available in the raw supplementary data. Note that Myoviridae, Siphoviridae, and Podoviridae are viral families of the Caudovirales order. Download FIG S5, JPG file, 1.5 MB. Copyright © 2022 Wittmers et al. 2022 Wittmers et al. https://creativecommons.org/licenses/by/4.0/ This content is distributed under the terms of the Creative Commons Attribution 4.0 International license.

**FIG 4:**
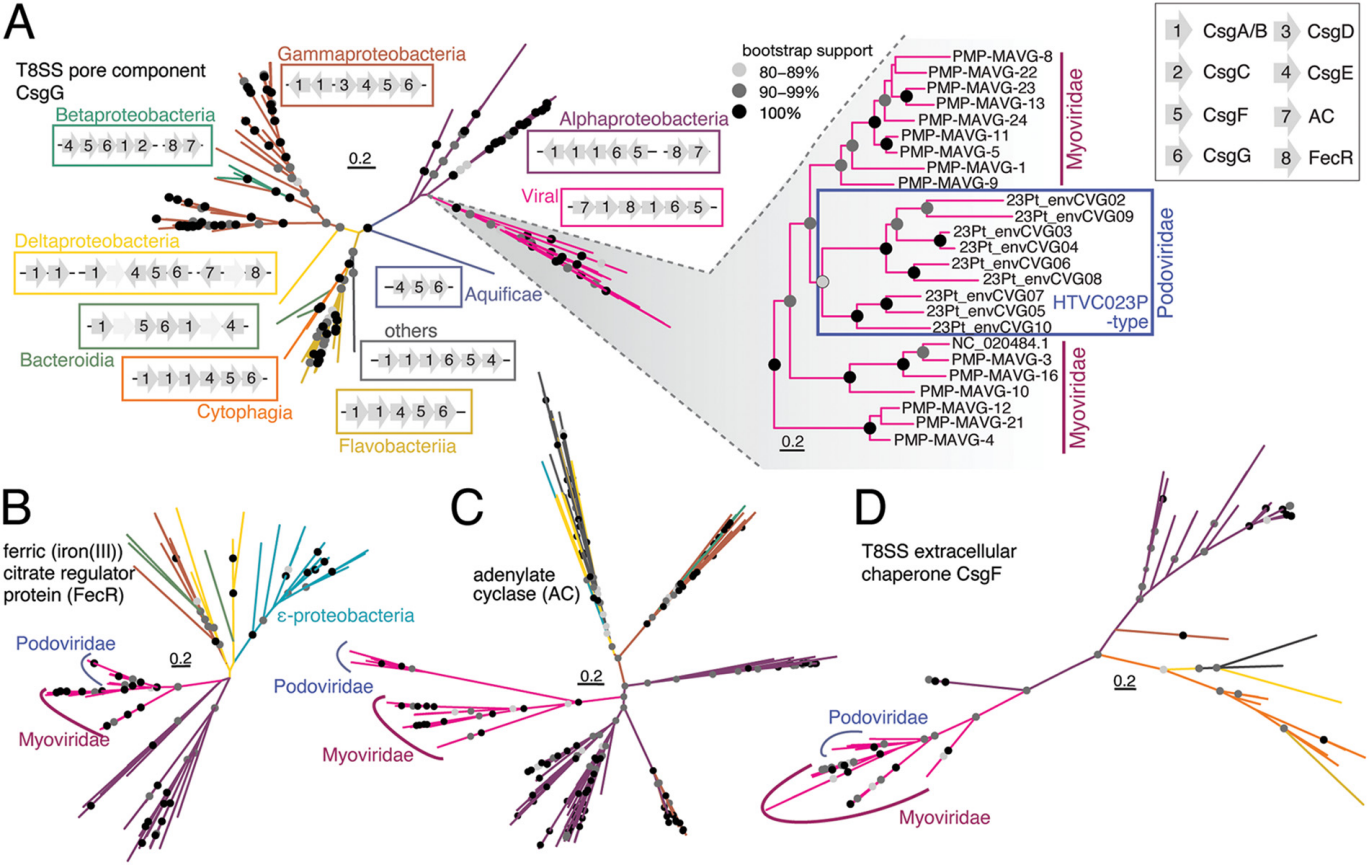
Evolutionary analyses point to cross-viral family T8SS gene transfer. (A–D) ML reconstructions of (A) CsgG, (B) FecR, (C) AC, and (D) CsgF, incorporating protein sequences from Myoviridae and Podoviridae Pelagiphages as well as bacterial and marine environmental genomes, computed under the LG+F+R7, LG+R7, LG+F+R8, and LG+F+R5 models, respectively, with 1,000 ultrafast bootstrap iterations. (A) The inset shows the viral CsgG topology at a higher resolution. The sequence labeled NC_020484 comes from cultured HTVC008M, which infects *Pelagibacter* (20). Examples of T8SS region organization in each lineage are also depicted (A). (B–D) The lineage coloring follows that established in (A).

We also performed phylogenetic reconstructions of the four T8SS partner-AMGs that were amenable to such analyses (i.e., excluding HK and CsgA/B, as they are short, diverged, and/or include paralogs). Statistical evidence was present in support of the Podoviridae and Myoviridae CsgG, CsgF, FecR, and AC sequences being most closely related to the alphaproteobacterial versions (Fig. 4A to D). Moreover, the Podoviridae versions collectively branched together, but typically within those of Myoviridae, suggesting a transfer from the Myoviridae to Podoviridae, based on available taxonomic sampling. The organization of the viral modules also resembled the organization of the modules found in Alphaproteobacteria, with the T8SS components found in other bacterial classes (CsgC, CsgD, and CsgE) not being present (31). Together, these lines of evidence suggest a cross- viral family transfer of the entire module, with an original acquisition from an Alphaproteobacterium.

### Global distribution and ecological importance of Pelagiphage T8SS.

**FIG 5:**
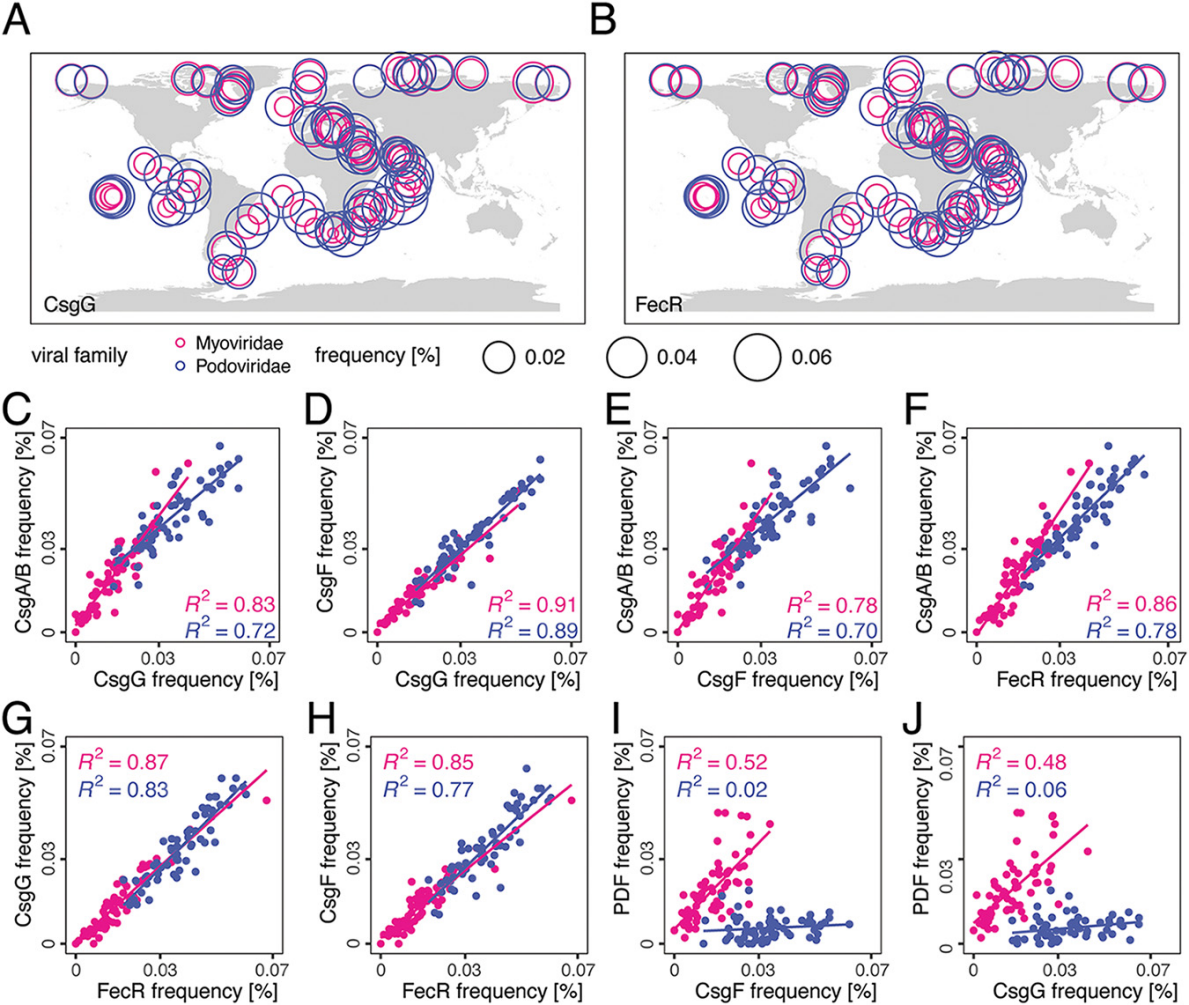
Consistent and abundant distribution of viral T8SS in the ocean. (A, B) Frequencies of (A) CsgG and (B) FecR at 65 GOV2.0 stations. (C–H) Comparison of frequencies for specific components from classical bacterial T8SS operons, as well as to viral FecR, at all 65 of the GOV2.0 stations analyzed. (I, J) or comparison we also show the frequency of T8SS components and the AMG peptide deformylase which are found in both Myoviridae and Podoviridae, but are not colocated with T8SS, and are not involved in similar functions. (A–J) Colors indicate Myoviridae (pink) and Podoviridae (blue) T8SS component frequencies, and all T8SS gene frequencies were normalized by total viral ORFs, per the respective ocean sampling station.

**FIG 6:**
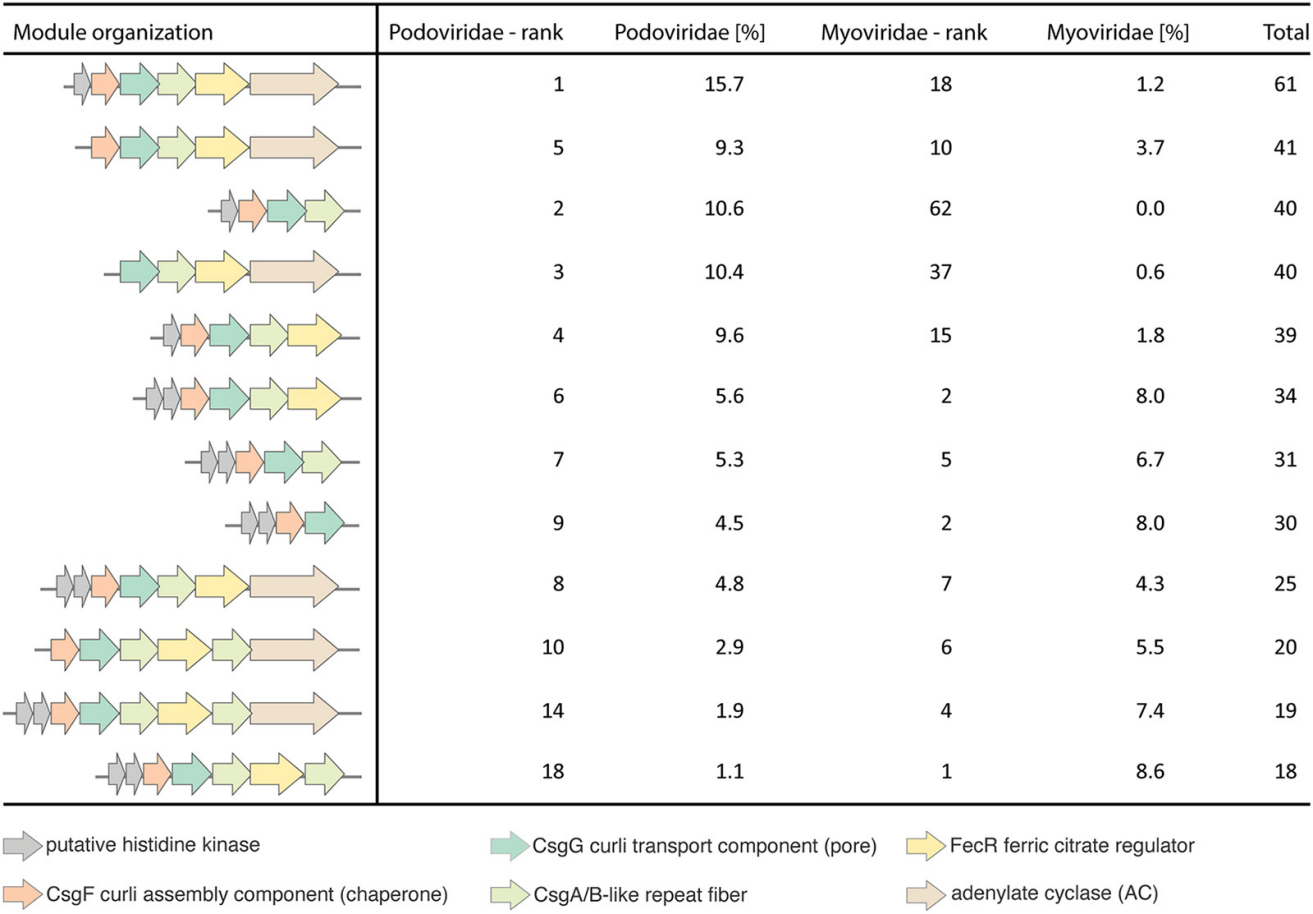
T8SS module organization varies slightly across modules recovered from all available viral contigs. Here, GOV2.0 (23) contigs of 10 kb or larger were analyzed, and the 10 most common compositions found in Podoviridae and Myoviridae, respectively, are depicted (see Data Set S1F for a full list). Podoviridae (%) and Myoviridae (%) indicate the percentage of this module found in sequences, relative to all modules from the respective viral family.

Beyond the Curli components identified in the envCVGs recovered herein, we sought to further characterize the distribution of the component parts from noncompletely assembled viruses in GOV2.0. Using this approach, viral Curli proteins were detected at all 65 GOV2.0 stations (Fig. 5A), as was the affiliated viral FecR (Fig. 5B). This demonstrated the widespread and frequent occurrence of phage Curli, which made up about 0.1% of all assembled viral proteins. We found evidence for expression of all of these in Pacific metatranscriptome data (32) collected from the location of the sorted 023Pt_pos29 reported herein, along a transect ending ~750 km offshore, as well as in a coastal metatranscriptome time-series (33) (Data Set S1B and C). The ratios in which the genes were found (pairwise average ratio of 0.93 ± 0.15) strongly suggests that the region (as represented in the envCVGs) is conserved across Pelagiphages in the ocean (Fig. 5C to J; Data Set S1F). Additional filtering to complete the Curli operons from GOV2.0 (but not the envCVGs) revealed that both the Myoviridae and Podoviridae T8SS typically had a similar organization to those identified in the envCVGs, except that the second copy of CsgA/B and AC were more frequently present in the Myoviridae (Fig. 6). Although FecR and AC are not always observed in the Curli regions, overall, the results suggest that selection is operating at a multigene level that includes the complete classical operon alongside viral FecR and AC.

## DISCUSSION

Previous studies have highlighted the diversity of Pelagiphage HTVC023P-type sequences in the ocean. Initially, a group of seven environmental contigs with PolA protein sequences were identified, and they appeared to be from abundant viruses, but with unknown hosts (34). This group was expanded through the GOV sequencing effort, comprising the two most globally distributed and abundant GOV clusters, VC_6 and VC_8 (35). The clusters were suggested to represent viruses of *Pelagibacter* upon the genome sequencing and analysis of the two first HTVC023P-type Pelagiphage isolates (6). Here, the completion of 80 Pelagiphage genomes from environmental data obviated biases potentially induced in phage cultivation efforts. Analysis of these genomes provided unprecedented insights into the genomic potential of HTVC023P-type phages, their AMGs, and partner-AMGs.

Phylogenetic and phylogenomic analyses revealed 10 statistically supported HTVCOP23P-type clades, not distinguished in prior reconstructions, including sister Clades IV and VI, for which potential hosts are less clear, although likely *Pelagibacter* or close relatives (Fig. 1A; Fig. S1). Among the HTVC023P-type clades, two contain isolated Pelagiphages as well as sequences from uncultivated phages (Clades I and II), and the other eight are formed of uncultivated members only. When we compared the phages captured herein to a large, well-characterized group of Myoviridae that infect marine Cyanobacteria (36), specifically *Synechococcus* or *Prochlorococcus*, we observed a similar degree of sequence diversity, highlighting the fact that phages with <50% AAI and sharing only 25% of their orthogroups can be associated with the same broad host lineages (Fig. S6).

10.1128/msystems.01522-21.6 FIG S6 Comparison of shared orthogroups (OGs) and amino acid identity for two widespread marine virus groups infecting different hosts. Specifically, the number of genes in OGs (as percentages) versus average amino acid identity (AAI) for members of those OGs in (A) HTVC023P-type Pelagiphages described herein and (B) Cyanomyophages infecting *Synechococcus* and *Prochlorococcus* from the genomes provided in (36). For (A, B), each dot represents a pairwise comparison between two genomes, including pairwise AAI (*y*-axis) and orthogroup overlap (*x*-axis). Percent shared OGs is defined by the total number of proteins found within two compared genomes that are part of an OG that is found in both genomes. Download FIG S6, EPS file, 2.1 MB. Copyright © 2022 Wittmers et al. 2022 Wittmers et al. https://creativecommons.org/licenses/by/4.0/ This content is distributed under the terms of the Creative Commons Attribution 4.0 International license.

Gene predictions for the HTCV023P-type Pelagiphage envCVGs rendered a greatly expanded gene catalog, with the pan-genome being 15-fold larger than previously reported (6). The recovery of considerable diversity, supported by complete genome sequences, also enabled the discovery of 41 AMGs not known in the HTVC023P-type Pelagiphages. Notably, due to the amount of microdiversity present in the Pelagiphages (26), it is likely that only a fraction of the AMGs present in nature. It is also possible that those obtained are found preferentially in the viruses recovered, due to the methods used. We expect that new efforts and techniques will lead to further representation of Pelagiphages. Here, the largest functional group of HTVC023P-type AMGs contained enzymes generally associated with DNA metabolism and modification. The same has been seen for both Cyanophage (9) and Pelagiphage (7) Myoviridae. Hence, the influences of phages on DNA metabolism and modification appear to be particularly important in the interactions between marine phages and their hosts.

Among the larger group of AMGs associated with nucleotide modification, a cluster involved in thymidine hypermodification of DNA stands out due to its high prevalence, along with that of a partner-AMG in Clade V, VIII, and IX Pelagiphages. The closest cultivated, non-*Pelagibacter*-infecting relatives to HTVC023P-like Podoviridae, Pseudomonas infecting M6-like phages, exhibit similar AMG partnering (30). In the M6-like system, the TS always occurs with an aG/PT-PPlase1 and is important for protecting viral DNA from host restriction enzymes via the process of thymidine hypermodifications, replacing 30 to 40 percent of thymidine bases with modified derivatives in Pseudomonas phage M6 and Salmonella phage Vil (30). As in the M6-like phages, the two proteins in the HTVC023P-type are located in close proximity to PolA, which assembles DNA nucleotides (Fig. 2C and 3A). The phylogenetic reconstruction of both TS and aG/PT-PPlase1 confirmed the relationship of the M6-/PaMx11-like and HTVC023P-type proteins, and for the TS, also that of Alphaproteobacteria phage phiJl001 (Fig. S5 and S6). In contrast to these Podoviridae, in Myoviridae that infect marine Cyanobacteria and others that infect *Pelagibacter*, the TS, which is not closely related to the M6-/PaMx11-like TS, occurs without aG/PT-PPlase1 and thus is presumed to only be involved in thymidylate synthesis (7, 30) (Fig. 3A). The HTVC023P-like Pelagiphage systems are also sometimes colocated with an additional 5′-deoxynucleotidase and a beta-glucosyl-HMC-alpha-glucosyl-transferase, which potentially broadens the functionality of the system beyond the scope previously reported for the Pseudomonas phages (30). Together, true functional characterization of at least some of these partner-AMGs in cultivated phages suggests a mechanism for considerably expanded resistance capabilities against host defenses involving digestion by restriction enzymes that is augmented in Clade V, VIII, and IX HTVC023P-type Pelagiphages.

Open ocean environments, such as those where *Pelagibacter* thrive, are known for oftentimes having a limited availability of nutrients, which has resulted in the genome streamlining of pelagic bacterial genomes (1) and presumably imposes restraints on the cost of a viral burden to a host (14). The fact that the partner-AMGs likely function as a collective raises questions about their acquisition and retention, including whether they might be derived from the same source, and about their importance to host ecology.

In the case of the TS and aG/PT-PPse1, these proteins appear possibly to be the products of vertical evolution after their original acquisition (Fig. S4 and S5). However, interpretation of the TS and aG/PT-PPse1 origins and transfers are not straightforward, given the paucity of nearby phages with hosts that inhabit the same environment and the paraphyletic nature of the viral region of the tree. Moreover, for both genes, the entire viral group is too divergent from cellular homologs to postulate on the bacterial lineage of origin, making it unclear whether they were acquired from the same organism. It might at first appear that the partner-AMGs involved in DNA hypermodification were present in multiple viral groups (post-acquisition), lost from HTVC023P-type Clades I, II, III, VII, and X, and retained by all HTVC023P-type Clade V, VIII and IX members. Alternatively, the partner-AMGs might have been acquired by a marine Myoviridae that is not present in current sequence databases or by one that infects SAR11 but has since lost the partner after a transfer to the last common ancestor of the Clade V, VIII and IX Podoviridae analyzed herein, with a global redistribution after the divergence of these clades.

Beyond the general pattern of AMGs putatively involved in nucleotide biosynthesis and modifications being common, our results revealed a high prevalence of complete T8SS in HTVC023P-type Pelagiphages. The T8SS involved the greatest number of colocated genes and retained a highly conserved operon-like structure in Pelagiphage from both the Podoviridae and Myoviridae phage families (Fig. 3A and B; Fig. 4; Fig. S3). Further, they appeared to encode all of the required components for a functional T8SS (Fig. 3C), although they were rewired relative to those in model Gammaproteobacteria, where T8SS have been best characterized (37, 38). Specifically, we identified genes encoding T8SS proteins CsgG and CsgF, which encode the Curli outer membrane (OM) pore and an associated extracellular chaperone, respectively, in bacteria (37). These two genes were both annotated in the first cultured Pelagimyoviridae genome (20) and were later identified in uncultivated Myoviridae Pelagiphages (7); the later study also inferred the possible presence of other T8SS genes, based on a gene co-occurrence network analysis. We explicitly identified the other essential T8SS proteins in the Myoviridae and Podoviridae, specifically the Curli fiber proteins (CsgA/B).

The T8SS module identified herein, in both the *Pelagibacter*-infecting Myoviridae and Podoviridae, highlight two striking aspects of the Pelagiphage T8SS that universally deviate from those in bacteria. The novel aspects of the Pelagiphage T8SS are especially notable, given that their host, *Pelagibacter*, does not encode any components. Thus, an altogether new function is imparted on the host upon infection. First, the Pelagiphage Curli regions lack the periplasmic secretion channel gate CsgE (37) (Fig. 3C). This suggests that the OM pore is either gated by a promiscuous host-encoded gate or remains bi-directionally open and allows the inward and/or outward flux of small macromolecules (38). Second, phage components AC, HK, and FecR are not part of canonical bacterial Curli operons. In model Gammaproteobacteria, AC production of cAMP leads to the transcription of the T8SS transcriptional regulator, CsgD, and interacts with a cognate sensor HK, resulting in enhanced biofilm development (21). Outside Gammaproteobacteria, the T8SS transcriptional regulator is unknown, as CsgD is typically lacking in most bacterial lineages (31) (Fig. 3A), as it is from the Pelagiphages studied herein. FecR is a regulator known for its role in inducing citrate-dependent Fe^3+^ transport in bacteria, which is notable here, given the importance of iron limitation in structuring surface ocean microbial communities. In model Gammaproteobacteria, CsgD also appears to interact with FecR (39). Hence, our results from across the envCVGs suggest an alternative route to T8SS regulation and function, relative to those well-studied in bacteria, that may additionally point to possible FecR T8SS regulatory roles in multiple bacterial lineages.

Although the precise origins of the viral T8SS module remain mysterious, and even more so for the other partner-AMG-types, for the four proteins where the phylogeny could be reconstructed, the Pelagiphage T8SS module components exhibited similar topologies. Specifically, they form a monophyletic clade for viral sequences from both families, suggesting transmission between them, and a likely origination from a more ancient transfer from an Alphaproteobacterium. These results seem to contrast with observations made for the Cyanophages, for which it has been shown that when multiple AMGs are involved in the same biochemical pathway (for example, cyanophage pentose phosphate pathway genes, transaldolase C, regulatory protein CP12, Zwf, and Gnd), they do not necessarily appear to have similar evolutionary histories (9, 19). Specifically, transaldolase C appears to have been passed among Myoviridae and Podoviridae infecting Cyanobacteria, with its cellular origin remaining unclear, while CP12 has, so far, only been observed in Myoviridae Cyanophages and was acquired from the host *Prochlorococcus.* Zwf was restricted to T4 Myoviruses infecting Cyanobacteria and was acquired from its host, while the origin of phage Gnd could not be reconstructed. This suggests that, in several cases, AMGs that ultimately function together have been acquired as single entities (19), similar to what has been reported for viruses of marine predatory protists (40, 41).

One potential explanation for the cross-family module transfer exhibited by Pelagiphage T8SS, and more tentatively, the TS and aG/PT-PPse1, is that it occurred during coinfection of the same host. Coinfection would result in a higher local concentration of phages than would occur in seawater (42, 43). Although recombination rates decline with increased evolutionary distance (50), observations herein support its occurrence as an important vector for the evolution of marine phages with overlapping host ranges. Its retention, given the burden it imposes, presumably provides some advantages to the host during infection (42). These advantages can be hypothesized based on their roles in other systems and on a consideration of *Pelagibacter* life history. First, the production of Curli by bacterial pathogens of animals (31, 38) can cause cell aggregation, thereby contributing to extracellular biofilms that limit phage penetration (44). In this context, Curli fiber production by infected *Pelagibacter* could protect cells from infection by competing viruses, thereby providing a defense against superinfection. Second, phage encounters with subsequent host cells might be augmented through host aggregation, a concept (called “sibling capture”) put forth previously when Curli components were observed in Pelagiphage Myoviridae (7). Alternatively, the T8SS channel could serve to import cofactors, such as cobalamin, thiamine-related compounds, or iron-containing siderophores (in line with the presence of FecR) that support aspects of *Pelagibacter* metabolism needed by the phage to support its replication. Finally, cell aggregation using virally encoded Curli fibers provides a plausible mechanism that could explain the high rates of parasexual intragenic recombination observed in *Pelagibacter* (26, 45, 46). Thus far, no mechanism has been proposed to explain how these planktonic organisms achieve one of the highest rates of recombination observed in bacteria (47). In the Curli sibling capture model we propose, a high *Pelagibacter* cell abundance, augmented by Curli-induced aggregation, would exponentially increase homologous recombination, which has been theorized to contribute to the success of *Pelagibacter* populations (47).

The apparent transfer of a multi-AMG region between distant lineages of viruses is reminiscent of classical theory on bacteriophage evolution, which proposed that phages are favorable combinations of gene modules, one for each function (e.g., a module for structural components), challenging the conventional definition of a “species” (48). However, genomic studies of isolated viruses have found that although genetic mosaicism exists, sometimes even between viral families, it is rare within the Caudovirales (49). In fact, in the Caudovirales, it is mainly observed in six highly mosaic phage genera (infecting Escherichia coli, Salmonella, *Bordetella*, Staphylococcus, and Mycobacterium) and rarely corrupts the vertical evolutionary signal (49). Nevertheless, the analysis of available phages as of 2016 (50) revealed varying levels of shared gene content between lytic phages (which had relatively low gene flow) and lysogenic phages (which had relatively high gene flow). Given that Pelagiphages have been shown to integrate (51), they may be expected to be among the phages more affiliated with high gene content flow. However, recombination events tend to occur between closely related phages with high nucleotide sequence similarity (interspecific recombination) (19, 52), but these phages are much more closely related than those transferring material herein. The degree that these patterns apply to Pelagiphages, or even to phages in general, is still unclear. Further, to our knowledge, no gene module transfer akin to our findings has been observed for viral AMGs. Regardless, our results demonstrate that, beyond structural components, multi-AMG exchanges can manifest as entire-module transfers between abundant marine phages.

### Conclusion.

Our studies reveal coherent, evolutionary distinct clades of HTVC023P-type Pelagiphages, which have, as a whole, been considered among the most abundant in the ocean (6, 22). The complete genomes presented contain multiple AMGs that are not present in cultivated HTVC023P-type Pelagiphages. The uncultivated Pelagiphages further highlight more complex gene acquisitions and retention that bring novel adaptations of consequence to host biology during infection, most notably partner-AMGs involved in DNA modifications and T8SS. These appear to be inherited among viral lineages that are independent of the known host lineage (as it exists in the modern day), with apparent transfer between viral families. Such multigene transfers between viral lineages have not previously been observed for AMGs. Additionally, they appear to be distinct from multiple independent acquisitions from hosts seen in other marine viruses (19) and different from the proposition and mechanisms behind the transfer of core-phage-gene modules (48). In the case of the T8SS, it brings a novel function to the host, *Pelagibacter*, presumably altering the host physiology during infection with the expression of Curli fibers and resulting conduit between the cell and the exterior environment. Moreover, all of the partner-AMG analyses and phylogenies placed multiple environmental sequences within the vicinity of those from the envCVGs presented herein, indicating that a breadth of HTCV023P-type diversity remains to be recovered. Our expanded view of the genomes of these viruses, including the identification and the recovery of clades with no prior genomic representation, provides insights into how HTVC023P-type Pelagiphages manipulate host metabolism and evolutionary power extending beyond being simple agents of mortality.

## MATERIALS AND METHODS

Samples were collected in Monterey Bay (11th April 2019, 36°44.695 N; 122°1.284 W) from a depth of 15 m. Cells were sorted using an Influx FACS, then frozen at −80°C. Afterwards, sorts were subjected to MDA, sequenced using paired-end Illumina HiSeq, and assembled using the methods described in (32). Viral contigs in assemblies were identified using VirSorter (53), and the circularity of viral contigs, indicating genome completeness, was tested using the minimus2 (54) pipeline (v1.5.5) with no contig merging and a 100 bp overlap required. Circularized genomes were retained. Then, ORFs were predicted using Prodigal (v2.6.3) (55) and, alongside those of two cultured HTVC023P-type Pelagiphages, used to iteratively query SAG data (24, 56, 57) and GOV2.0 (23) using hmmscan (v3.1b2; ‘-E 1e-04’) (58) with the HMM model for DNA polymerase A (PolA; PF00476). Identified contigs that were larger than 10 kb were processed further, and ORFs were predicted as above. Taxonomic predictions for bacterial contigs in the published SAG (24), where we recovered an HTVC023P-type Pelagiphage (herein named 023Pt_pos29), was made using CAT (v5.11) (45) with diamond (v0.9.34) after making ORF predictions as above. 023Pt_aos01 was finished manually by identifying repetitive gene fragments at contig ends. The circularity of GOV2.0 was tested as described above. Note that a recent publication reported a SAG (vSAG 37-F6; 13 kb) belonging to HTVC023P-type Pelagiphages (26). We did not include this SAG in our analyses (phylogenetic and orthogroup/partner) because its incompleteness meant that it did not have most of the relevant genes. A comparison of the capsid protein, which was present in the partial assembly of vSAG37-F6, showed an 83% aa similarity with that of HTVC027P, and vSAG37-F6 was previously identified as a HTVC023P-type phage (6). Those genomes having ≥45% PolA aa similarity to the cultured Pelagiphages were then analyzed further. Specifically, orthologous proteins (either within viral genomes or between viral genomes) were identified using OrthoFinder (v2.2.6) with the default settings (59). For the identification of AMGs, ORFs were annotated with BLASTP against nr (E value ≤ 10^−10^) and hmmscan against Pfam (same E value cutoff). All ORFs having functional/taxonomic information (BLASTP) or a functional annotation (Pfam) were then checked manually for homology to proteins in Bacteria, Archaea, or Eukaryotes. This manual curation enabled the screening for those mistakenly identified as AMGs due to viral contamination in database environmental sequences/MAGs. Annotations based on TARA Ocean MAGs (60) were excluded due to the widespread contamination of genomes with viral sequences. In addition, pairwise average amino acid identity was calculated using the aai.rb ruby script from the enveomics code collection (61).

We performed phylogenetic and phylogenomic analyses of virally encoded proteins. First, we constructed a PolA phylogenetic tree that consisted of diverse representative sequences of the PolA family as previously described (6), along with those from the novel genomes identified herein. Sequences were aligned with MAFFT (v7.455) G-INS-i using the VSM option (–unalignlevel 0.6) to control over-alignment (62). Ambiguously aligned positions were trimmed using trimAl (63). Synechococcus_phage_S-RIP1 (ANW82925.1) and Roseobacter_phage_CRP-5 (QBQ72679.1) were removed due to incomplete PolA protein sequences. The best fitting substitution models, here and below, were predicted using ModelFinder in IQ-TREE (v0.1.6) (64, 65). A maximum-likelihood (ML) tree was then inferred with 1,000 ultrafast bootstrap replicates (here and below). Phylogenetic trees were visualized in R using the ggtree package (66, 67). Biogeographic provinces were assigned based on the provinces defined in (25). All figures, including heatmaps, were visualized using ggplot2 (68). Maps were visualized in R using the simple features (sf) package (69) extensions to ggplot2 with vectorized maps from the rnaturalearth data package (70).

The multigene phylogenetic reconstruction of Pelagiphages HTVC023P and HTVC027P and related environmental phage genomes was based on PolA, DEAD/DEAH box helicase, primase, capsid, and one hypothetical protein (e.g., HTVC023P-type cultured representative HTVC027P: QGZ17791.1, QGZ17868.1, QGZ17785.1, QGZ17856.1, QGZ17840.1). Sequences were manually inspected, and paralogs were removed. The refined sequences were aligned as above and trimmed. Individual protein alignments were then concatenated, resulting in a 2,480 aa alignment. The LG+I+G4+F model was used alongside an empirical mixture model with 20 profiles to test a better statistical fit (LG+C20+I+G+F) while maintaining the same matrix (LG), amino acid frequency computation, and gamma categories as in the optimized model. The tree was rooted with Pseudomonas phage PaMx11 and Alphaproteobacteria phage phiiJL001 as an outgroup during visualization.

We used the more conserved viral T8SS region proteins, which were amenable to robust phylogenetic analysis, that we had predicted from the phage genomes (i.e., CsgG, CsgF, FecR, and AC) to query 2,873 NCBI bacterial reference genomes and JGI/IMG data, specifically, 4,931 marine bacterial and archaeal genomes (categorized as Ocean, Coastal, Pelagic, or Neritic), 128 bacterial genomes identified as “endosymbionts” or “intracellular”, and 12,714 marine SAGs (19). Using recovered BLASTP hits (E value < 10^−10^), we constructed a preliminary alignment, computed with MAFFT (‘–auto’). By manual inspection, partial sequences covering 50% or less of the alignment were removed, and split genes were merged. CsgG paralogs missing the highly conserved CsgG motifs (a second copy of CsgG, mainly in Gammaproteobacteria) and FecR paralogs (mainly in Alphaproteobacteria) were removed. The filtered alignment was realigned using MAFFT (‘–linsi’), and the alignment was trimmed. The respective ML trees were computed under the LG+F+R7 (CsgG), LG+F+R8 (AC), LG+R7 (FecR), and LG+F+R5 (CsgF) models with 1,000 ultrafast bootstrap replicates. The bacterial taxonomy was based on NCBI taxonomy IDs. Both the HK and the CsgA/B sequences were too divergent from cellular homologs to derive phylogenetic reconstructions. Similarly, for the reconstruction of the TS and aG/PT-PPlase1 protein sequences, we queried the Global Earth Microbiome database (71) and NCBI nr with the same BLASTP cutoffs as used above. Alignments were constructed using MAFFT (‘–auto’) and trimmed as described above, with both ML trees being computed under the LG+R10 model with 1,000 ultrafast bootstrap replicates in IQ-TREE (65).

Metatranscriptomes from the North Pacific (32) were queried using all proteins from a total of 29 viral Curli-encoding Pelagiphage genomes and TBLASTN queries (100% nucleotide identity threshold) (Data Set S1B). GOV2.0 was queried separately for CsgG, CsgF, CsgA/B, and FecR with BLASTP (E value < 10^−10^), using the viral homologs from both the Podoviridae and Myoviridae complete genome sequences. For each, the number of hits per station was normalized by the total number of viral ORFs, and each was assigned to the Myoviridae or Podoviridae family based on the viral family of the best BLAST hit to the query sequences. Hits for which the identity was lower than the lowest identity observed within each protein and family combination of query sequences with known taxonomic affiliation (i.e., from envCVGs and cultured Pelagiphages) were excluded in order to retain only sequences from Myoviridae and Podoviridae Pelagiphages. The same GOV2.0 protein query hits were used to identify and pull contigs carrying T8SS components from GOV2.0. Contigs were excluded from further analysis when a T8SS component was at a terminal position to avoid the consideration of incomplete regions.

### Data availability.

Alignments and trees are available via FigShare (https://figshare.com/projects/Worden_Lab_-_curli_operon_transfer/94439). Reads for 23Pt_envCVGpos01 FACS-derived Pelagiphage (PRJNA699323) as well as the genome and annotations (MW574966) are available in GenBank. Gene predictions and annotations for other circularized phage genomes (https://figshare.com/projects/Worden_Lab_-_curli_operon_transfer/94439).Metatranscriptomes are deposited under PRJNA464924 to PRJNA464930 (26).

## References

[1] Giovannoni SJ. 2017. SAR11 bacteria: the most abundant plankton in the oceans. Annu Rev Mar Sci 9:231–255. doi:10.1146/annurev-marine-010814-015934. 27687974

[2] Giovannoni SJ, Britschgi TB, Moyer CL, Field KG. 1990. Genetic diversity in Sargasso Sea bacterioplankton. Nature 345:60–63. doi:10.1038/345060a0. 2330053

[3] Rappé MS, Connon SA, Vergin KL, Giovannoni SJ. 2002. Cultivation of the ubiquitous SAR11 marine bacterioplankton clade. Nature 418:630–633. doi:10.1038/nature00917. 12167859

[4] Monaghan EA, Freel KC, Rappé MS, Bowman J. 2020. Isolation of SAR11 marine bacteria from cryopreserved seawater. mSystems 5:e00954-20. doi:10.1128/mSystems.00954-20. 33361323 PMC7762794

[5] Buchholz HH, Michelsen ML, Bolaños LM, Browne E, Allen MJ, Temperton B. 2021. Efficient dilution-to-extinction isolation of novel virus–host model systems for fastidious heterotrophic bacteria. ISME J 15:1585–1598. doi:10.1038/s41396-020-00872-z. 33495565 PMC8163748

[6] Zhang Z, Qin F, Chen F, Chu X, Luo H, Zhang R, Du S, Tian Z, Zhao Y. 2021. Culturing novel and abundant pelagiphages in the ocean. Environ Microbiol 23:1145–1161. doi:10.1111/1462-2920.15272. 33047445

[7] Zaragoza-Solas A, Rodriguez-Valera F, López-Pérez M. 2020. Metagenome mining reveals hidden genomic diversity of pelagimyophages in aquatic environments. mSystems 5:e00905-19. doi:10.1128/mSystems.00905-19. 32071164 PMC7029224

[8] Warwick-Dugdale J, Buchholz HH, Allen MJ, Temperton B. 2019. Host-hijacking and planktonic piracy: how phages command the microbial high seas. Virol J 16:15. doi:10.1186/s12985-019-1120-1. 30709355 PMC6359870

[9] Thompson LR, Zeng Q, Libusha KHHKUSA, JoAnne SWCS. 2011. Phage auxiliary metabolic genes and the redirection of cyanobacterial host carbon metabolism. Proc Natl Acad Sci USA 108:E757–E764. doi:10.1073/pnas.1102164108. 21844365 PMC3182688

[10] Hurwitz BL, U'Ren JM. 2016. Viral metabolic reprogramming in marine ecosystems. Curr Opin Microbiol 31:161–168. doi:10.1016/j.mib.2016.04.002. 27088500

[11] Breitbart M. 2012. Marine viruses: truth or dare. Annu Rev Mar Sci 4:425–448. doi:10.1146/annurev-marine-120709-142805. 22457982

[12] Kieft K, Zhou Z, Anderson RE, Buchan A, Campbell BJ, Hallam SJ, Hess M, Sullivan MB, Walsh DA, Roux S, Anantharaman K. 2021. Ecology of inorganic sulfur auxiliary metabolism in widespread bacteriophages. Nat Commun 12:3503. doi:10.1038/s41467-021-23698-5. 34108477 PMC8190135

[13] Mann NH, Cook A, Millard A, Bailey S, Clokie M. 2003. Bacterial photosynthesis genes in a virus. Nature 424:741. doi:10.1038/424741a. 12917674

[14] Breitbart M, Thompson LR, Suttle CA, Sullivan MB. 2007. Exploring the vast diversity of marine viruses. Oceanog 20:135–139. doi:10.5670/oceanog.2007.58.

[15] Chénard C, Suttle CA. 2008. Phylogenetic diversity of sequences of Cyanophage photosynthetic gene psbA in marine and freshwaters. Appl Environ Microbiol 74:5317–5324. doi:10.1128/AEM.02480-07. 18586962 PMC2546643

[16] Lindell D, Sullivan MB, Johnson ZI, Tolonen AC, Rohwer F, Chisholm SW. 2004. Transfer of photosynthesis genes to and from Prochlorococcus viruses. Proc Natl Acad Sci USA 101:11013–11018. doi:10.1073/pnas.0401526101. 15256601 PMC503735

[17] Sullivan MB, Lindell D, Lee JA, Thompson LR, Bielawski JP, Chisholm SW. 2006. Prevalence and evolution of core photosystem II genes in marine cyanobacterial viruses and their hosts. PLoS Biol 4:e234. doi:10.1371/journal.pbio.0040234. 16802857 PMC1484495

[18] Lindell D, Jaffe JD, Johnson ZI, Church GM, Chisholm SW. 2005. Photosynthesis genes in marine viruses yield proteins during host infection. Nature 438:86–89. doi:10.1038/nature04111. 16222247

[19] Ignacio-Espinoza JC, Sullivan MB. 2012. Phylogenomics of T4 cyanophages: lateral gene transfer in the ‘core’ and origins of host genes. Environ Microbiol 14:2113–2126. doi:10.1111/j.1462-2920.2012.02704.x. 22348436

[20] Zhao Y, Temperton B, Thrash JC, Schwalbach MS, Vergin KL, Landry ZC, Ellisman M, Deerinck T, Sullivan MB, Giovannoni SJ. 2013. Abundant SAR11 viruses in the ocean. Nature 494:357–360. doi:10.1038/nature11921. 23407494

[21] Hufnagel DA, Evans ML, Greene SE, Pinkner JS, Hultgren SJ, Chapman MR. 2016. The catabolite repressor protein-cyclic AMP complex regulates csgD and biofilm formation in uropathogenic Escherichia coli. J Bacteriol 198:3329–3334. doi:10.1128/JB.00652-16. 27698083 PMC5116936

[22] Martinez-Hernandez F, Fornas Ò, Lluesma Gomez M, Garcia-Heredia I, Maestre-Carballa L, López-Pérez M, Haro-Moreno JM, Rodriguez-Valera F, Martinez-Garcia M. 2019. Single-cell genomics uncover Pelagibacter as the putative host of the extremely abundant uncultured 37-F6 viral population in the ocean. ISME J 13:232–236. doi:10.1038/s41396-018-0278-7. 30228380 PMC6299107

[23] Gregory AC, Tara Oceans Coordinators, Zayed AA, Conceição-Neto N, Temperton B, Bolduc B, Alberti A, Ardyna M, Arkhipova K, Carmichael M, Cruaud C, Dimier C, Domínguez-Huerta G, Ferland J, Kandels S, Liu Y, Marec C, Pesant S, Picheral M, Pisarev S, Poulain J, Tremblay J-É, Vik D, Acinas SG, Babin M, Bork P, Boss E, Bowler C, Cochrane G, de Vargas C, Follows M, Gorsky G, Grimsley N, Guidi L, Hingamp P, Iudicone D, Jaillon O, Kandels-Lewis S, Karp-Boss L, Karsenti E, Not F, Ogata H, Pesant S, Poulton N, Raes J, Sardet C, Speich S, Stemmann L, Sullivan MB, Sunagawa S, Wincker P, Babin M, Bowler C, Culley AI, de Vargas C, Dutilh BE, Iudicone D, Karp-Boss L, Roux S, Sunagawa S, Wincker P, Sullivan MB. 2019. Marine DNA viral macro- and microdiversity from pole to pole. Cell 177:1109–1123.e14. doi:10.1016/j.cell.2019.03.040. 31031001 PMC6525058

[24] Pachiadaki MG, Brown JM, Brown J, Bezuidt O, Berube PM, Biller SJ, Poulton NJ, Burkart MD, La Clair JJ, Chisholm SW, Stepanauskas R. 2019. Charting the complexity of the marine microbiome through single-cell genomics. Cell 179:1623–1635.e11. doi:10.1016/j.cell.2019.11.017. 31835036 PMC6919566

[25] Longhurst AR. 2007. Ecological geography of the sea. Elsevier.

[26] Martinez-Hernandez F, Diop A, Garcia-Heredia I, Bobay L-M, Martinez-Garcia M. 2022. Unexpected myriad of co-occurring viral strains and species in one of the most abundant and microdiverse viruses on Earth. ISME J 16:1025–1035. doi:10.1038/s41396-021-01150-2. 34775488 PMC8940918

[27] Giglione C, Vallon O, Meinnel T. 2003. Control of protein life-span by N-terminal methionine excision. EMBO J 22:13–23. doi:10.1093/emboj/cdg007. 12505980 PMC140049

[28] Jia B, Jia X, Kim KH, Jeon CO. 2017. Integrative view of 2-oxoglutarate/Fe(II)-dependent oxygenase diversity and functions in bacteria. Biochim Biophys Acta Gen Subj 1861:323–334. doi:10.1016/j.bbagen.2016.12.001. 27919802

[29] Small SK, Puri S, O'Brian MR. 2009. Heme-dependent metalloregulation by the iron response regulator (Irr) protein in Rhizobium and other Alpha-proteobacteria. Biometals 22:89–97. doi:10.1007/s10534-008-9192-1. 19093075 PMC2659648

[30] Lee Y-J, Dai N, Walsh SE, Müller S, Fraser ME, Kauffman KM, Guan C, Corrêa IR, Weigele PR. 2018. Identification and biosynthesis of thymidine hypermodifications in the genomic DNA of widespread bacterial viruses. Proc Natl Acad Sci USA 115:E3116–E3125. doi:10.1073/pnas.1714812115. 29555775 PMC5889632

[31] Dueholm MS, Albertsen M, Otzen D, Nielsen PH. 2012. Curli functional amyloid systems are phylogenetically widespread and display large diversity in operon and protein structure. PLoS One 7:e51274. doi:10.1371/journal.pone.0051274. 23251478 PMC3521004

[32] Needham DM, Poirier C, Hehenberger E, Jiménez V, Swalwell JE, Santoro AE, Worden AZ. 2019. Targeted metagenomic recovery of four divergent viruses reveals shared and distinctive characteristics of giant viruses of marine eukaryotes. Philos Trans R Soc B 374:20190086. doi:10.1098/rstb.2019.0086. PMC6792449 31587639

[33] Nowinski B, Smith CB, Thomas CM, Esson K, Marin R, Preston CM, Birch JM, Scholin CA, Huntemann M, Clum A, Foster B, Foster B, Roux S, Palaniappan K, Varghese N, Mukherjee S, Reddy TBK, Daum C, Copeland A, Chen I-MA, Ivanova NN, Kyrpides NC, Glavina del Rio T, Whitman WB, Kiene RP, Eloe-Fadrosh EA, Moran MA. 2019. Microbial metagenomes and metatranscriptomes during a coastal phytoplankton bloom. Sci Data 6:129. doi:10.1038/s41597-019-0132-4. 31332186 PMC6646334

[34] Schmidt HF, Sakowski EG, Williamson SJ, Polson SW, Wommack K. 2014. Shotgun metagenomics indicates novel family A DNA polymerases predominate within marine virioplankton. ISME J 8:103–114. doi:10.1038/ismej.2013.124. 23985748 PMC3869006

[35] Roux S, Tara Oceans Coordinators, Brum JR, Dutilh BE, Sunagawa S, Duhaime MB, Loy A, Poulos BT, Solonenko N, Lara E, Poulain J, Pesant S, Kandels-Lewis S, Dimier C, Picheral M, Searson S, Cruaud C, Alberti A, Duarte CM, Gasol JM, Vaqué D, Bork P, Acinas SG, Wincker P, Sullivan MB, Coordinators TO. 2016. Ecogenomics and potential biogeochemical impacts of globally abundant ocean viruses. Nature 537:689–693. doi:10.1038/nature19366. 27654921

[36] Crummett LT, Puxty RJ, Weihe C, Marston MF, Martiny JBH. 2016. The genomic content and context of auxiliary metabolic genes in marine cyanomyoviruses. Virology 499:219–229. doi:10.1016/j.virol.2016.09.016. 27693926

[37] Taylor J, Matthews S. 2015. New insight into the molecular control of bacterial functional amyloids. Front Cell Infect Microbiol 5:33–37. doi:10.3389/fcimb.2015.00033. 25905048 PMC4389571

[38] Evans ML, Chapman MR. 2014. Curli biogenesis: order out of disorder. Biochim Biophys Acta 1843:1551–1558. doi:10.1016/j.bbamcr.2013.09.010. 24080089 PMC4243835

[39] Brombacher E, Dorel C, Zehnder AJB, Landini P. 2003. The curli biosynthesis regulator CsgD co-ordinates the expression of both positive and negative determinants for biofilm formation in Escherichia coli. Microbiology (Reading) 149:2847–2857. doi:10.1099/mic.0.26306-0. 14523117

[40] Needham DM, Yoshizawa S, Hosaka T, Poirier C, Choi CJ, Hehenberger E, Irwin NAT, Wilken S, Yung C-M, Bachy C, Kurihara R, Nakajima Y, Kojima K, Kimura-Someya T, Leonard G, Malmstrom RR, Mende DR, Olson DK, Sudo Y, Sudek S, Richards TA, DeLong EF, Keeling PJ, Santoro AE, Shirouzu M, Iwasaki W, Worden AZ. 2019. A distinct lineage of giant viruses brings a rhodopsin photosystem to unicellular marine predators. Proc Natl Acad Sci USA 116:20574–20583. doi:10.1073/pnas.1907517116. 31548428 PMC6789865

[41] Fischer MG, Kelly I, Foster LJ, Suttle CA. 2014. The virion of Cafeteria roenbergensis virus (CroV) contains a complex suite of proteins for transcription and DNA repair. Virology 466–467:82–94. doi:10.1016/j.virol.2014.05.029. 24973308

[42] Kauffman KM, Chang WK, Brown JM, Hussain FA, Yang JY, Polz MF, Kelly L. 2022. Resolving the structure of phage-bacteria interactions in the context of natural diversity. Nature Communications 2022.06.27.449121. https://www.nature.com/articles/s41467-021-27583-z. 10.1038/s41467-021-27583-z PMC8766483 35042853

[43] Kupczok A, Neve H, Huang KD, Hoeppner MP, Heller KJ, Franz CMAP, Dagan T. 2018. Rates of mutation and recombination in Siphoviridae phage genome evolution over three decades. Mol Biol Evol 35:1147–1159. doi:10.1093/molbev/msy027. 29688542 PMC5913663

[44] Vidakovic L, Singh PK, Hartmann R, Nadell CD, Drescher K. 2018. Dynamic biofilm architecture confers individual and collective mechanisms of viral protection. Nat Microbiol 3:26–31. doi:10.1038/s41564-017-0050-1. 29085075 PMC5739289

[45] Vergin KL, Tripp HJ, Wilhelm LJ, Denver DR, Rappé MS, Giovannoni SJ. 2007. High intraspecific recombination rate in a native population of Candidatus Pelagibacter ubique (SAR11). Environ Microbiol 9:2430–2440. doi:10.1111/j.1462-2920.2007.01361.x. 17803769

[46] Vos M, Didelot X. 2009. A comparison of homologous recombination rates in bacteria and archaea. ISME J 3:199–208. doi:10.1038/ismej.2008.93. 18830278

[47] Giovannoni S, Temperton B, Zhao Y. 2013. Giovannoni et al. reply. Nature 499:E4–E5. doi:10.1038/nature12388. 23887435

[48] Botstein D. 1980. A theory of modular evolution for bacteriophages. Ann N Y Acad Sci 354:484–491. doi:10.1111/j.1749-6632.1980.tb27987.x. 6452848

[49] Low SJ, Džunková M, Chaumeil P-A, Parks DH, Hugenholtz P. 2019. Evaluation of a concatenated protein phylogeny for classification of tailed double-stranded DNA viruses belonging to the order Caudovirales. Nat Microbiol 4:1306–1315. doi:10.1038/s41564-019-0448-z. 31110365

[50] Mavrich TN, Hatfull GF. 2017. Bacteriophage evolution differs by host, lifestyle and genome. Nat Microbiol 2:17112. doi:10.1038/nmicrobiol.2017.112. 28692019 PMC5540316

[51] Zhao Y, Qin F, Zhang R, Giovannoni SJ, Zhang Z, Sun J, Du S, Rensing C. 2019. Pelagiphages in the Podoviridae family integrate into host genomes. Environ Microbiol 21:1989–2001. doi:10.1111/1462-2920.14487. 30474915

[52] Pope WH, Bowman CA, Russell DA, Jacobs-Sera D, Asai DJ, Cresawn SG, Jacobs WR, Jr, Hendrix RW, Lawrence JG, Hatfull GF, Science SEAPHAG and E, Education PHIR and, Course MG. 2015. Whole genome compa proteinsrison of a large collection of mycobacteriophages reveals a continuum of phage genetic diversity. Elife 4:e06416. doi:10.7554/eLife.06416. 25919952 PMC4408529

[53] Roux S, Enault F, Hurwitz BL, Sullivan MB. 2015. VirSorter: mining viral signal from microbial genomic data. PeerJ 3:e985. doi:10.7717/peerj.985. 26038737 PMC4451026

[54] Hunt M, Gall A, Ong SH, Brener J, Ferns B, Goulder P, Nastouli E, Keane JA, Kellam P, Otto TD. 2015. IVA: accurate de novo assembly of RNA virus genomes. Bioinformatics 31:2374–2376. doi:10.1093/bioinformatics/btv120. 25725497 PMC4495290

[55] Hyatt D, Chen G-L, LoCascio PF, Land ML, Larimer FW, Hauser LJ. 2010. Prodigal: prokaryotic gene recognition and translation initiation site identification. BMC Bioinformatics 11:119. doi:10.1186/1471-2105-11-119. 20211023 PMC2848648

[56] Tsementzi D, Wu J, Deutsch S, Nath S, Rodriguez-R LM, Burns AS, Ranjan P, Sarode N, Malmstrom RR, Padilla CC, Stone BK, Bristow LA, Larsen M, Glass JB, Thamdrup B, Woyke T, Konstantinidis KT, Stewart FJ. 2016. SAR11 bacteria linked to ocean anoxia and nitrogen loss. Nature 536:179–183. doi:10.1038/nature19068. 27487207 PMC4990128

[57] Thompson LR, Haroon MF, Shibl AA, Cahill MJ, Ngugi DK, Williams GJ, Morton JT, Knight R, Goodwin KD, Stingl U. 2019. Red Sea SAR11 and *Prochlorococcus* single-cell genomes reflect globally distributed pangenomes. Appl Environ Microbiol 85:e00369-19. doi:10.1128/AEM.00369-19. 31028022 PMC6581160

[58] Finn RD, Clements J, Eddy SR. 2011. HMMER web server: interactive sequence similarity searching. Nucleic Acids Res 39:W29–W37. doi:10.1093/nar/gkr367. 21593126 PMC3125773

[59] Emms DM, Kelly S. 2019. OrthoFinder: phylogenetic orthology inference for comparative genomics. Genome Biol 20:238. doi:10.1186/s13059-019-1832-y. 31727128 PMC6857279

[60] Tully BJ, Graham ED, Heidelberg JF. 2018. The reconstruction of 2,631 draft metagenome-assembled genomes from the global oceans. Sci Data 5:170203. doi:10.1038/sdata.2017.203. 29337314 PMC5769542

[61] Rodriguez-R LM, Konstantinidis KT. 2016. The enveomics collection: a toolbox for specialized analyses of microbial genomes and metagenomes. PeerJ Preprints doi:10.7287/peerj.preprints.1900v1.

[62] Katoh K, Standley DM. 2016. A simple method to control over-alignment in the MAFFT multiple sequence alignment program. Bioinformatics 32:1933–1942. doi:10.1093/bioinformatics/btw108. 27153688 PMC4920119

[63] Capella-Gutierrez S, Silla-Martinez JM, Gabaldon T. 2009. trimAl: a tool for automated alignment trimming in large-scale phylogenetic analyses. Bioinformatics 25:1972–1973. doi:10.1093/bioinformatics/btp348. 19505945 PMC2712344

[64] Kalyaanamoorthy S, Minh BQ, Wong TKF, von Haeseler A, Jermiin LS. 2017. ModelFinder: fast model selection for accurate phylogenetic estimates. Nat Methods 14:587–589. doi:10.1038/nmeth.4285. 28481363 PMC5453245

[65] Minh BQ, Schmidt HA, Chernomor O, Schrempf D, Woodhams MD, von Haeseler A, Lanfear R. 2020. IQ-TREE 2: new models and efficient methods for phylogenetic inference in the genomic era. Mol Biol Evol 37:1530–1534. doi:10.1093/molbev/msaa015. 32011700 PMC7182206

[66] Ihaka R, Gentleman R. 1996. R: a language for data analysis and graphics. J Computational and Graphical Statistics 5:299–314. doi:10.1080/10618600.1996.10474713.

[67] Yu G, Smith DK, Zhu H, Guan Y, Lam TT-Y. 2017. ggtree: an r package for visualization and annotation of phylogenetic trees with their covariates and other associated data. Methods Ecol Evol 8:28–36. doi:10.1111/2041-210X.12628.

[68] Wickham H. 2011. ggplot2. Wires Comp Stat 3:180–185. doi:10.1002/wics.147.

[69] Pebesma EJ. 2018. Simple features for R: standardized support for spatial vector data. R. J 10:439. doi:10.32614/RJ-2018-009.

[70] Kelso NV, Patterson T. 2010. Introducing natural earth data-naturalearthdata. Com Geographia Technica 5:25.

[71] Nayfach S, IMG/M Data Consortium, Roux S, Seshadri R, Udwary D, Varghese N, Schulz F, Wu D, Paez-Espino D, Chen I-M, Huntemann M, Palaniappan K, Ladau J, Mukherjee S, Reddy TBK, Nielsen T, Kirton E, Faria JP, Edirisinghe JN, Henry CS, Jungbluth SP, Chivian D, Dehal P, Wood-Charlson EM, Arkin AP, Tringe SG, Visel A, Abreu H, Acinas SG, Allen E, Allen MA, Alteio LV, Andersen G, Anesio AM, Attwood G, Avila-Magaña V, Badis Y, Bailey J, Baker B, Baldrian P, Barton HA, Beck DAC, Becraft ED, Beller HR, Beman JM, Bernier-Latmani R, Berry TD, Bertagnolli A, Bertilsson S, Bhatnagar JM, Bird JT, Blanchard JL, Blumer-Schuette SE, Bohannan B, Borton MA, Brady A, Brawley SH, Brodie J, Brown S, Brum JR, Brune A, Bryant DA, Buchan A, Buckley DH, Buongiorno J, Cadillo-Quiroz H, Caffrey SM, Campbell AN, Campbell B, Carr S, Carroll J, Cary SC, Cates AM, Cattolico RA, Cavicchioli R, Chistoserdova L, Coleman ML, Constant P, Conway JM, Mac Cormack WP, Crowe S, Crump B, Currie C, Daly R, DeAngelis KM, Denef V, Denman SE, Desta A, Dionisi H, Dodsworth J, Dombrowski N, Donohue T, Dopson M, Driscoll T, Dunfield P, Dupont CL, Dynarski KA, Edgcomb V, Edwards EA, Elshahed MS, Figueroa I, Flood B, Fortney N, Fortunato CS, Francis C, Gachon CMM, Garcia SL, Gazitua MC, Gentry T, Gerwick L, Gharechahi J, Girguis P, Gladden J, Gradoville M, Grasby SE, Gravuer K, Grettenberger CL, Gruninger RJ, Guo J, Habteselassie MY, Hallam SJ, Hatzenpichler R, Hausmann B, Hazen TC, Hedlund B, Henny C, Herfort L, Hernandez M, Hershey OS, Hess M, Hollister EB, Hug LA, Hunt D, Jansson J, Jarett J, Kadnikov VV, Kelly C, Kelly R, Kelly W, Kerfeld CA, Kimbrel J, Klassen JL, Konstantinidis KT, Lee LL, Li W-J, Loder AJ, Loy A, Lozada M, MacGregor B, Magnabosco C, Maria da Silva A, McKay RM, McMahon K, McSweeney CS, Medina M, Meredith L, Mizzi J, Mock T, Momper L, Moran MA, Morgan-Lang C, Moser D, Muyzer G, Myrold D, Nash M, Nesbø CL, Neumann AP, Neumann RB, Noguera D, Northen T, Norton J, Nowinski B, Nüsslein K, O’Malley MA, Oliveira RS, Maia de Oliveira V, Onstott T, Osvatic J, Ouyang Y, Pachiadaki M, Parnell J, Partida-Martinez LP, Peay KG, Pelletier D, Peng X, Pester M, Pett-Ridge J, Peura S, Pjevac P, Plominsky AM, Poehlein A, Pope PB, Ravin N, Redmond MC, Reiss R, Rich V, Rinke C, Rodrigues JLM, Rodriguez-Reillo W, Rossmassler K, Sackett J, Salekdeh GH, Saleska S, Scarborough M, Schachtman D, Schadt CW, Schrenk M, Sczyrba A, Sengupta A, Setubal JC, Shade A, Sharp C, Sherman DH, Shubenkova OV, Sierra-Garcia IN, Simister R, Simon H, Sjöling S, Slonczewski J, Correa de Souza RS, Spear JR, Stegen JC, Stepanauskas R, Stewart F, Suen G, Sullivan M, Sumner D, Swan BK, Swingley W, Tarn J, Taylor GT, Teeling H, Tekere M, Teske A, Thomas T, Thrash C, Tiedje J, Ting CS, Tully B, Tyson G, Ulloa O, Valentine DL, Van Goethem MW, VanderGheynst J, Verbeke TJ, Vollmers J, Vuillemin A, Waldo NB, Walsh DA, Weimer BC, Whitman T, van der Wielen P, Wilkins M, Williams TJ, Woodcroft B, Woolet J, Wrighton K, Ye J, Young EB, Youssef NH, Yu FB, Zemskaya TI, Ziels R, Woyke T, Mouncey NJ, Ivanova NN, Kyrpides NC, Eloe-Fadrosh EA. 2021. A genomic catalog of Earth’s microbiomes. Nat Biotechnol 39:499–509. doi:10.1038/s41587-020-0718-6. 33169036 PMC8041624

[72] Yan Z, Yin M, Chen J, Li X. 2020. Assembly and substrate recognition of curli biogenesis system. Nat Commun 11:241. doi:10.1038/s41467-019-14145-7. 31932609 PMC6957492

[73] Coutinho FH, Cabello-Yeves PJ, Gonzalez-Serrano R, Rosselli R, López-Pérez M, Zemskaya TI, Zakharenko AS, Ivanov VG, Rodriguez-Valera F. 2020. New viral biogeochemical roles revealed through metagenomic analysis of Lake Baikal. Microbiome 2020.04.02.019802. https://microbiomejournal.biomedcentral.com/articles/10.1186/s40168-020-00936-4. 10.1186/s40168-020-00936-4 PMC7678222 33213521

