# Supplementary figures and images for: Genomes from Uncultivated Pelagiphages Reveal Multiple Phylogenetic Clades Exhibiting Extensive Auxiliary Metabolic Genes and Cross-Family Multigene Transfers
Source: mSystems. 2022 Aug 16;7(5):e01522-21. doi: 10.1128/msystems.01522-21 (PMC9599517; doi:10.1128/msystems.01522-21)

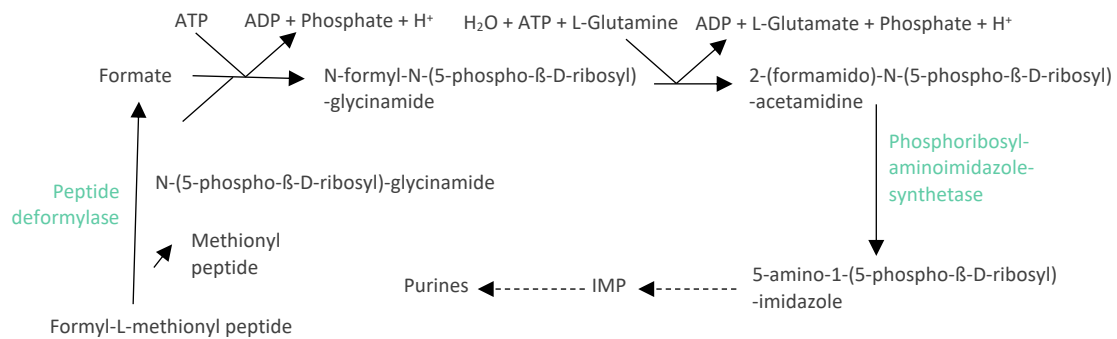

Supplement: FIG S2 [file msystems.01522-21-s0002.pdf]

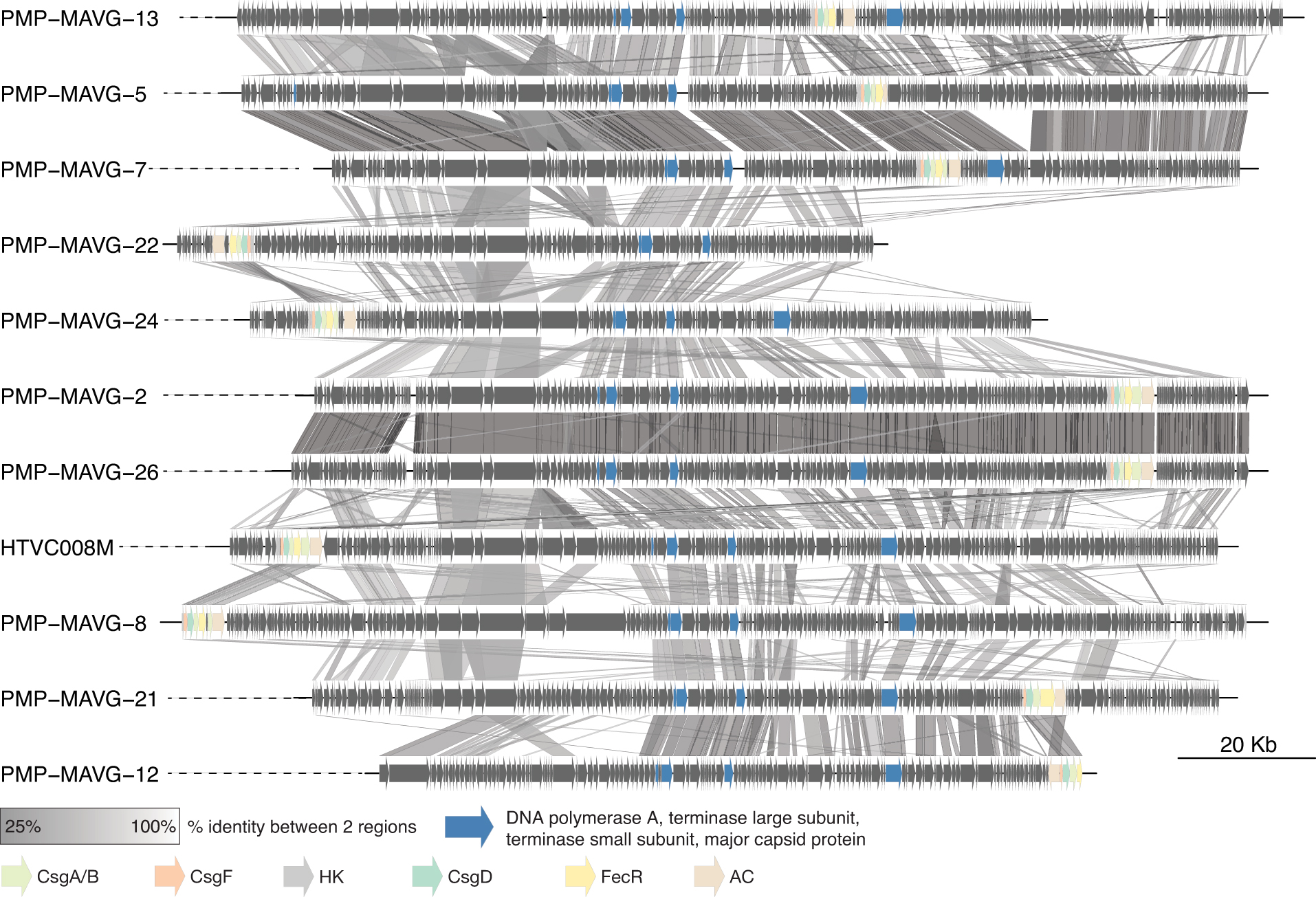

Supplement: FIG S3 [file msystems.01522-21-s0003.jpg]

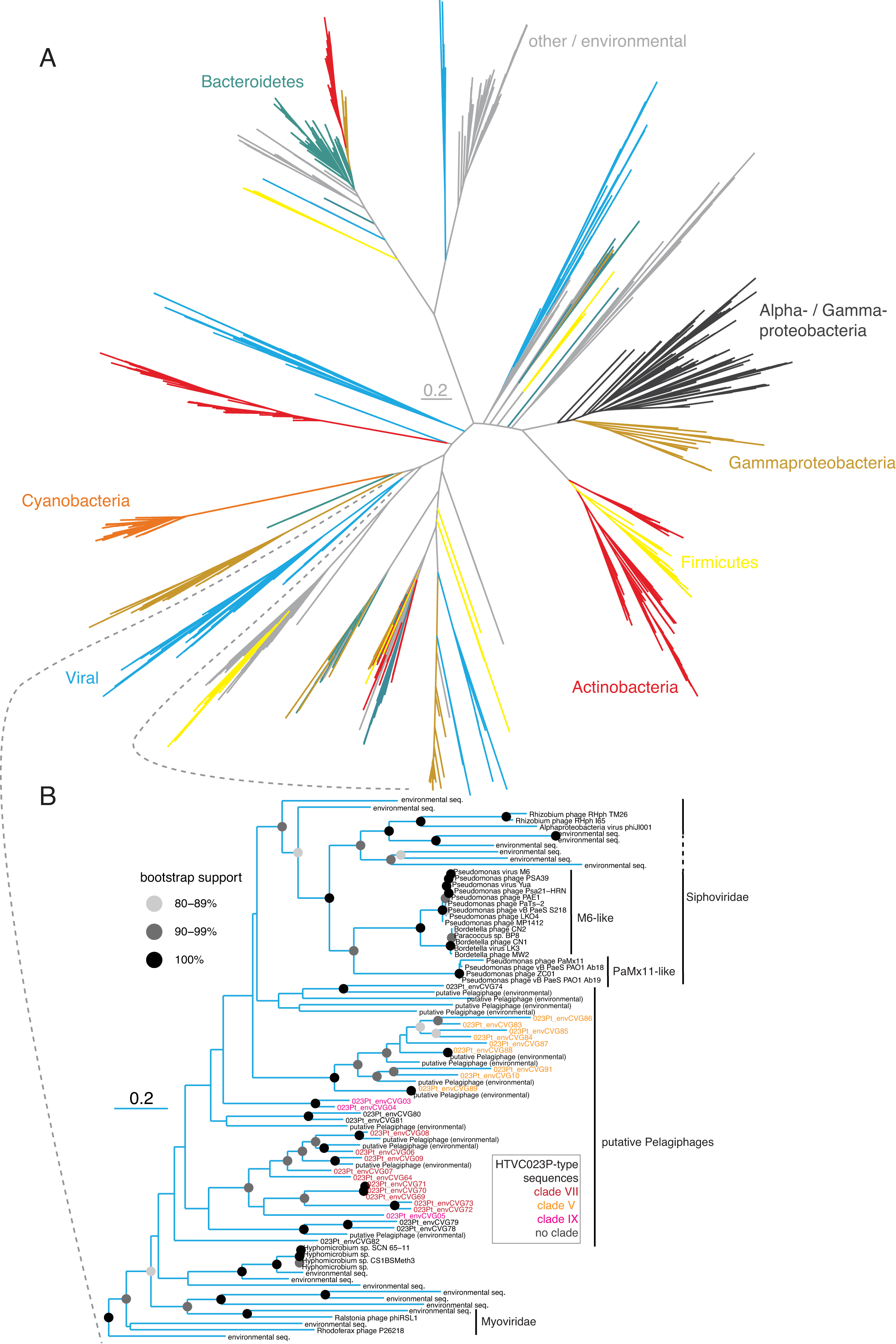

Supplement: FIG S5 [file msystems.01522-21-s0005.jpg]
